# Elimination Resistance: Characterizing Multi-compartment Toxicokinetics of the Neonicotinoid Thiacloprid in the Amphipod *Gammarus pulex* Using Bioconcentration and Receptor-Binding Assays

**DOI:** 10.1021/acs.est.3c01891

**Published:** 2023-06-07

**Authors:** Johannes Raths, Linda Schinz, Annika Mangold-Döring, Juliane Hollender

**Affiliations:** † Department of Environmental Chemistry, Swiss Federal Institute of Aquatic Science and Technology—Eawag, Überlandstrasse 133, 8600 Dübendorf, Switzerland; ‡ Institute of Biogeochemistry and Pollutant Dynamics, ETH Zürich, Universitätstrasse 16, 8092 Zürich, Switzerland; § Department of Aquatic Ecology and Water Quality Management, Wageningen University, P.O. Box 47, 6700 Wageningen, The Netherlands

**Keywords:** bioaccumulation, invertebrates, micropollutants, organic contaminants, insecticides

## Abstract

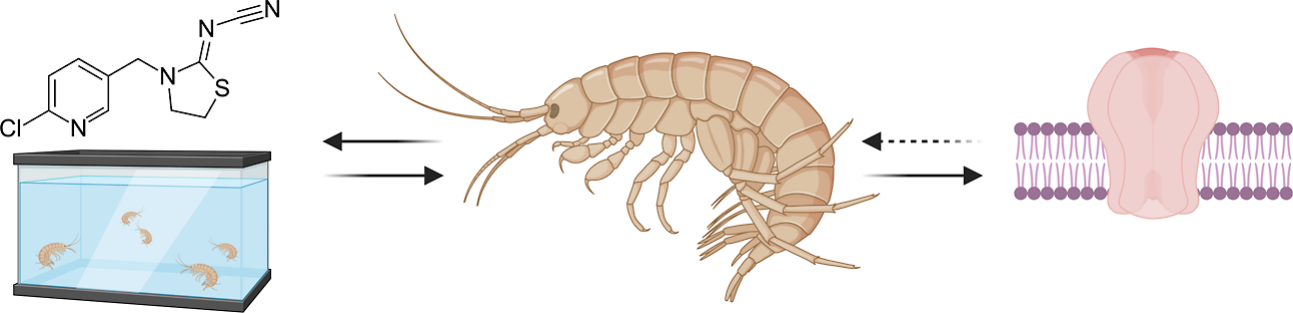

Delayed toxicity is a phenomenon observed for aquatic invertebrates exposed to nicotinic acetylcholine receptor (nAChR) agonists, such as neonicotinoids. Furthermore, recent studies have described an incomplete elimination of neonicotinoids by exposed amphipods. However, a mechanistic link between receptor binding and toxicokinetic modeling has not been demonstrated yet. The elimination of the neonicotinoid thiacloprid in the freshwater amphipod *Gammarus pulex* was studied in several toxicokinetic exposure experiments, complemented with in vitro and in vivo receptor-binding assays. Based on the results, a two-compartment model was developed to predict the uptake and elimination kinetics of thiacloprid in *G. pulex*. An incomplete elimination of thiacloprid, independent of elimination phase duration, exposure concentrations, and pulses, was observed. Additionally, the receptor-binding assays indicated irreversible binding of thiacloprid to the nAChRs. Accordingly, a toxicokinetic-receptor model consisting of a structural and a membrane protein (including nAChRs) compartment was developed. The model successfully predicted internal thiacloprid concentrations across various experiments. Our results help in understanding the delayed toxic and receptor-mediated effects toward arthropods caused by neonicotinoids. Furthermore, the results suggest that more awareness toward long-term toxic effects of irreversible receptor binding is needed in a regulatory context. The developed model supports the future toxicokinetic assessment of receptor-binding contaminants.

## Abstract 2

In the present study, we present a multi-compartment toxicokinetic modeling approach to evaluate the bioaccumulation of environmental contaminants (i.e., neonicotinoids) that are elimination resistant due to protein or receptor binding.

## Introduction

Neonicotinoids are one of the most widely applied classes of insecticides globally,^1,2^ with seven of them being commercially available worldwide: imidacloprid, thiacloprid, thiamethoxam, clothianidin, acetamiprid, nitenpyram, and dinotefuran (molecular structures are provided in Supporting Information A1).^2,3^ However, the widespread use and toxicity of neonicotinoid insecticides toward numerous non-target insect species, particularly pollinators,^4,5^ resulted in several bans of these insecticides in the last decade (i.e., imidacloprid, clothianidin, thiamethoxam, and thiacloprid for outdoor usage in the EU).^6^ Nevertheless, neonicotinoids are still extensively used in most other countries, such as the USA^7^ and China.^8^ Furthermore, the butenolide insecticide flupyradifurone, which potentially exerts less toxicity toward pollinators but has a similar mode of action, was introduced as a replacement candidate in 2015.^9^

**Figure 1:**
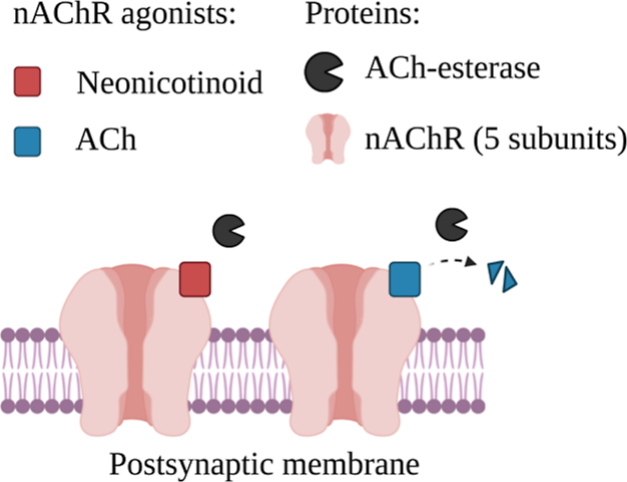
Illustration of the competition of neonicotinoids and ACh for nAChRs in the synaptic cleft. The hydrolysis of ACh by ACh-esterase is visualized with a dotted arrow. The nAChR is composed of five subunits and corresponding potential agonist-binding sites.

Several properties of neonicotinoids contributed to their worldwide adoption and versatility, replacing more problematic insecticides such as carbamates and organophosphates.^10^ Neonicotinoids are persistent, water-soluble, systemic, and highly selective insecticides with low toxicity and bioaccumulation potential in vertebrates.^3,11^ Neonicotinoids interfere with neural transmission in the central nervous system of invertebrates (Figure 1). They act as (partial) agonists of the nicotinic acetylcholine receptors (nAChRs) and compete with the endogenous neurotransmitter acetylcholine (ACh). In contrast to ACh, neonicotinoids are not hydrolyzed by acetylcholine esterase, leading to their prolonged action at the nAChRs.^12^ This interference causes continuous activation of the nAChRs, ultimately resulting in symptoms of neurotoxicity, such as paralysis. Differences in the subunits of the receptors in vertebrates and arthropod species result in a much stronger affinity and subsequent toxicity of neonicotinoids toward arthropods, with insects being the most sensitive class.^3,13^

Besides pollinators, neonicotinoids also affect aquatic organisms as they can reach surface waters through spray drift or run-off events.^5^ Since neonicotinoids are systemic insecticides designed for fast uptake and distribution in plants, contaminated plant material may be another route of neonicotinoid exposure.^14,15^ Recent studies have shown that the pollution levels of neonicotinoids in water bodies often exceed the environmental quality standards,^5,16^ provoking mostly chronic adverse effects on aquatic, non-target arthropods. Both acute and delayed toxicity,^17^ as well as long recovery times (i.e., 10 weeks up to more than 7 months),^18−20^ have been observed in thiacloprid-exposed aquatic invertebrates, such as *Gammarus pulex* (Linnaeus, 1758), during laboratory and mesocosm experiments. Gammarids are frequently used in aquatic monitoring studies^21,22^ due to their ecological importance for detritus decomposition, trophic transfer of nutrients and contaminants,^23^ and widespread occurrence.^21,24^ They are also typical non-target organisms and can take up contaminants through the gills (respiration) or their diet (i.e., contaminated leaves).^21^

The enrichment of a substance from the water phase into an organism is called bioconcentration.^25^ In the regulatory registration process of chemicals, bioconcentration factors (BCFs) and toxicokinetic rates are commonly determined in uptake and elimination experiments with fish, according to OECD 305.^25^ Due to ethical considerations, analogously designed studies for invertebrates were proposed as an alternative.^26,27^ Commonly, toxicokinetic parameters are derived using a simplified one-compartment approach.^28^ However, exploring the kinetics of certain compounds requires more complex (i.e., two-compartment) approaches to be sufficiently captured.^28−30^ Such is the case for neonicotinoids, as recent studies indicate that these substances are not completely eliminated from amphipods in neither laboratory^31,32^ nor field^22^ environments.

We hypothesized that the irreversible binding to the nAChRs causes the elimination resistance. For testing this hypothesis, we characterized the non-eliminating fraction of thiacloprid in*G. pulex* by performing several uptake-elimination experiments with a prolonged elimination phase and different exposure concentrations and exposure patterns (i.e., pulsed exposure). We selected thiacloprid because it was still permitted for use in Switzerland in 2019 and was found in field experiments in gammarids even when no thiacloprid was detected in the surface water.^22^ The relevance of binding to nAChRs for the elimination resistance of thiacloprid was evaluated by performing both in vivo and in vitro nAChR-binding assays. Thereby, irreversible binding was defined as no measurable depletion on the experimental time scale of up to 8 days. Eventually, based on the combined results of the uptake-elimination experiments and receptor-binding assays, a toxicokinetic model was developed to account for compounds with specific receptor-binding properties and to help understand long-term toxic effects caused by irreversible binding.

## Materials and Methods

### Test Animals

Specimens of *G. pulex* were collected from an uncontaminated creek^32,34^ near Zurich (Mönchaltdorfer Aa, 47.2749 °N, 8.7892 °E), located in a landscape conservation area. The sampling and experimental timeline covered the months of October (kinetic experiment I and concentration dependence II and III), November (pulsed exposure IV), and January (in vivo and in vitro receptor-binding assays V and VI) at water temperatures of 12, 7, and 1 °C, respectively. Genetic specifications of the population are provided elsewhere.^32^ Gammarids were kept in artificial pond water (APW)^33^ with a pH of 7.9 and at 15.5 °C. Details on the acclimation procedure are provided in Supporting Information A2. Lipid content was determined gravimetrically (Supporting Information A3).^32^ Data for lipid, protein, and thiacloprid contents are reported on a wet weight basis but can be converted to a dry weight basis using an experimentally determined factor of 5.4.^32^

### Toxicokinetic Experiments

Toxicokinetic experiments consisted of an uptake phase, where gammarids were exposed to test medium containing thiacloprid, followed by an elimination phase, where gammarids were transferred to medium without thiacloprid. The exposure medium was renewed every 5 days. All experiments were conducted in aerated 6 L glass tanks filled with APW, if not specified otherwise. A water temperature of 15.5 ± 1 °C and a 12:12 h light–dark cycle were maintained during the experiments. Gammarids were only fed during the elimination phase to avoid the sorption of thiacloprid to leaves and subsequent dietary uptake of sorbed thiacloprid. General experimental designs, as well as exposure times and concentrations, are displayed in Table 1.

**Table 1:** Overview of the Duration of the Different Toxicokinetic Experiments and Test Concentrations

no.	experiment	exposure [d]	elimination [d]	thiacloprid [μg L^–1^]
I	kinetic experiment	2	8	50 (200 nM)
II	concentration dependence—low	20, 20, 4	5, 5, 4	0.05, 0.5, 5
III	concentration dependence—high	2	2	5, 50, 500, 1500, 5000
IV	pulsed exposure^a^	2	3	5, 50
V	in vivo receptor assay	2	2	50
VI	in vitro receptor assay	b	b	b

a Three subsequent sequences of exposure and elimination were applied.

b Test conditions are specified in the in vitro receptor-binding section.

In order to confirm the previously observed incomplete elimination of thiacloprid from amphipods,^32^ a kinetic experiment with a prolonged elimination phase was performed (Table 1, I). Samples were taken in duplicate at regular time intervals.

To test a potential concentration dependence of thiacloprid bioconcentration (i.e., due to a maximal binding capacity), gammarids were exposed to seven different thiacloprid concentrations (Table 1, II and III). The exposure and elimination times were chosen to guarantee steady state conditions of both exposure and elimination. However, no steady state was reached during the uptake phase of the 0.05 μg L^–1^ exposure. Gammarids were sampled in triplicate at the end of the exposure and elimination phases. Additionally, samples for more time points (every 5 days) were taken during the exposure to 0.5 and 0.05 μg L^–1^ in order to obtain kinetic data for the toxicokinetic-receptor model.

To evaluate whether the residual thiacloprid body burden increases when gammarids are repeatedly exposed to thiacloprid, gammarids were exposed to three consecutive pulses of thiacloprid (Table 1, IV). During each of the three pulses, gammarids were sampled in triplicate on days 1, 2, 3, and 5 of each pulse.

Supplementing toxicokinetic experiments, including investigations on thiacloprid sorption to the exoskeleton and the contribution of physiological activity (i.e., respiration) to toxicokinetics using dead gammarids, are described in Supporting Information A4 and A10.

Gammarids that died during the experiments were removed from the test system and not sampled. Shortly before the start of each experiment, (<1 h) control samples of medium and gammarids were taken. Gammarids for tissue analysis (4 per replicate) were collected, rinsed with nanopure water, dry blotted, weighed (wet weight), snap frozen in liquid nitrogen, and stored at −20 °C until extraction.

### Sample Preparation

Tissue extracts were prepared by liquid extraction, as described elsewhere.^34^ First, 300 mg of 1 mm zirconia/silica beads (BioSpec Products, Inc.), 100 μL of internal standard (ISTD, 250 μg L^–1^ thiacloprid-*d*_4_ in methanol), and 500 μL of methanol were added. Then, samples were homogenized using a tissue homogenizer (2 × 15 s at 6 m s^–1^; FastPrep, MP Biomedicals) before centrifugation (10,000*g*, 6 min, 4 °C). The supernatant was collected using syringes and filtered through 0.45 μm regenerated cellulose filters (BGB Analytic AG). Filters were washed with 400 μL of pure methanol, and the two collected filtrates were combined. Medium samples (500 μL) were collected from the tanks, spiked with 100 μL of ISTD, and mixed with 400 μL of methanol.

### Membrane Protein Isolation and in Vivo Receptor-Binding Assay

Total protein and membrane protein (MP) content was determined based on isolation methods adapted from Maloney et al.^35^ The workflow is illustrated in Supporting Information A5. In brief, gammarid samples were taken shortly before (<1 h) the corresponding toxicokinetic experiments started, dry blotted on paper tissue, weighed, and snap frozen in liquid nitrogen. Samples were homogenized using approximately 300 mg of 1 mm zirconia/silica beads (pre-cooled 4 °C, BioSpec Products, Inc.) with a pre-cooled tissue lyser (15 s, 6 s^–1^, 4 °C; Bead Ruptor Elite, OMNI International). Next, dissociation medium (DM) was added (1 mL/per sample, 4 °C) to the tubes, and samples were homogenized again. The DM consisted of a buffer of 20 mM sodium phosphate and 150 mM sodium chloride (pH 7.0), as well as 0.1 mM phenylmethylsulfonyl fluoride (PMSF), 1 mM ethylenediaminetetraacetic acid (EDTA), 0.33 mg L^–1^ pepstatin, 0.33 mg L^–1^ chymostatin, and 0.33 mg L^–1^ leupeptin for protease inhibition. Samples were centrifuged (30 min, 1000*g*, 4 °C), and the supernatant (SN1) was collected with a pipette. The pellet was resuspended in DM (1 mL/sample, 4 °C) and centrifuged again (10 min, 1000*g*, 4 °C), and the supernatant (SN2) was combined with SN1 in 8 mL ultracentrifuge vials. A subsample of the combined supernatants was used for the determination of the total protein content. Afterward, the volume was adjusted to 7 mL using cold DM, and samples were ultracentrifuged (43,000*g*, 30 min, 4 °C, Ultracentrifuge CP100NX, Hitachi). Subsequently, the supernatant was carefully removed with a pipette, and the pellet was resuspended (MP extract) in 4 mL of DM. The concentrations of proteins in the supernatant and MP extract were quantified using the Pierce BCA Protein Assay Kit (Thermo Fisher Scientific) and calculated as described in Supporting Information A5.

**Figure 2:**
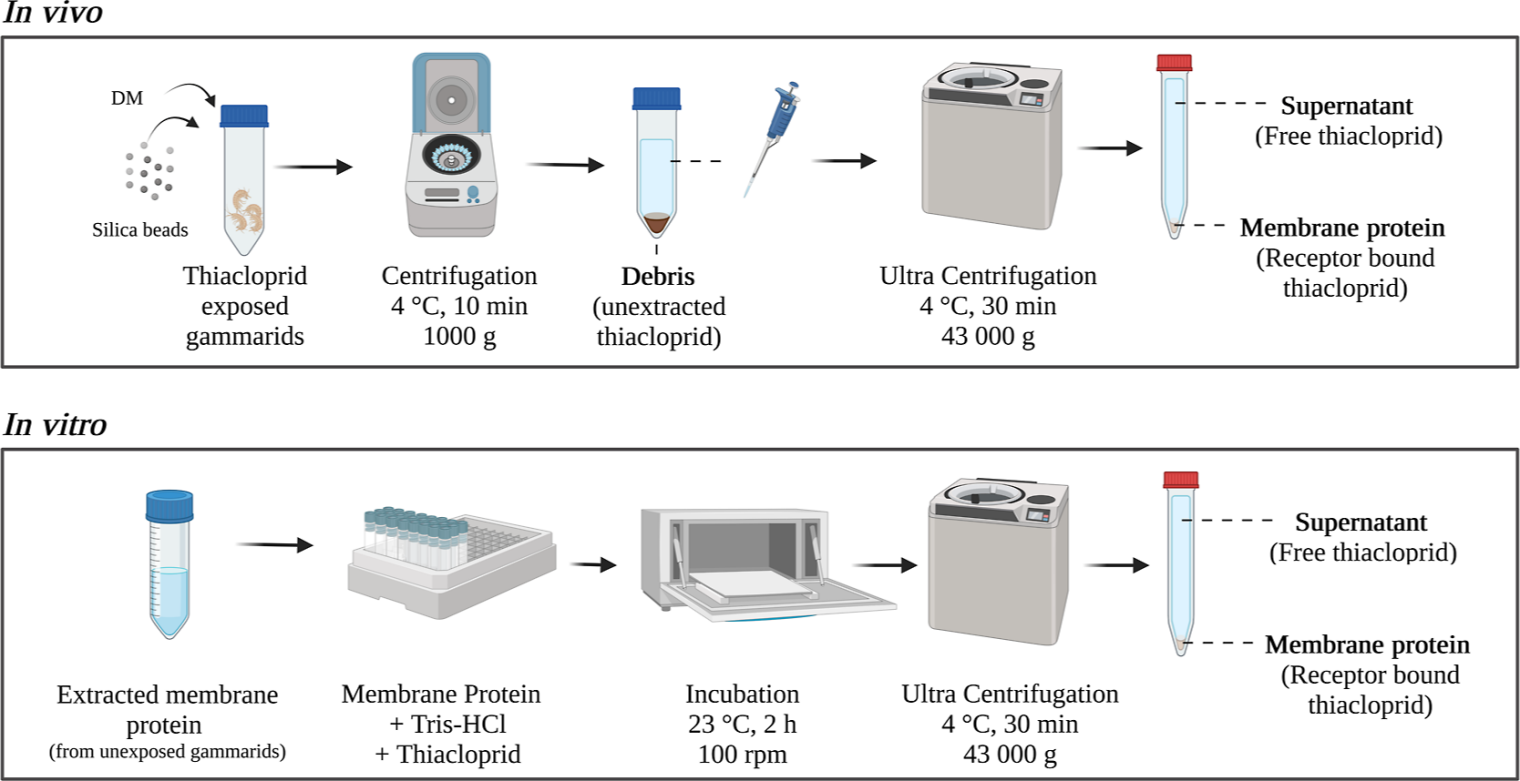
Two performed nAChR-binding approaches (in vivo: top, in vitro: bottom). DM = dissociation medium. The fractions analyzed by online-SPE LC-HRMS/MS (debris, supernatant, membrane protein) are indicated by bold letters.

For the in vivo receptor assay, exposed gammarids (Table 1, V) were sampled at the end of the exposure and at the end of the elimination phase (12 replicates each). Six replicates were extracted using the liquid extraction method for the determination of total thiacloprid content (recovery control). The other six replicates (fractionation) were treated as follows (Figure 2): membrane protein extraction was performed as described above. Afterward, the MP pellet was extracted for bound thiacloprid with 900 μL of MeOH after the addition of 100 μL ISTD and filtered through 0.45 μm cellulose filters. Additionally, thiacloprid was extracted from the debris (particles at the bottom after centrifugation at 1000*g*) and in the supernatant (after ultracentrifugation at 43,000*g*). The debris was extracted using methanol as described for gammarid tissue extractions. The supernatant was sampled like the medium samples (see sample preparation). The measured thiacloprid concentrations were normalized to the total body weight.

### In Vitro Receptor-Binding Assay

The in vitro nAChR binding assay was performed based on the methods described by Maloney et al.^35^ The methods were adapted to our facilities and to the use of non-radioactively labeled thiacloprid (Figure 2). The receptor-binding assay was prepared by combining 0.27 mg of MP (0.5 mL of MP extract) and 6 mL of Tris–HCl buffer (10 mM, pH = 7.4) spiked with thiacloprid in 8 mL ultracentrifuge vials. Final concentrations of thiacloprid in the vials corresponded to 0, 2.5, 5, 10, 25, and 50 nM. Each concentration was tested in four technical replicates split over multiple runs of the assay (8 vials each). After incubation for 2 h at 23 °C and 100 rpm, samples were ultracentrifuged (43,000*g*, 30 min, 4 °C). The incubation medium was sampled by combining 500 μL of the supernatant with 400 μL of MeOH and 100 μL of ISTD. The rest of the supernatant was removed, the pellet was resuspended in 6.5 mL of Tris–HCl, and the suspension was ultracentrifuged again. Subsequently, the supernatant was removed, and the pellet was extracted for thiacloprid concentration, as described for gammarid tissue extractions. Each assay included a protein recovery control in order to correct the measured receptor-bound amount of thiacloprid for the MP lost during the extraction steps.

### Chemical Analysis

All collected samples were stored at −20 °C until chemical analysis. Chemical analysis was performed using an automated online solid phase extraction system coupled with reversed phase (C18 column, Atlantis T3, 5 μm, 3 × 150 mm) liquid chromatography and high-resolution tandem mass spectrometry (online-SPE-LC-HRMS/MS) using the Orbitrap technology (Thermo Fisher Scientific Inc.). Ionization was performed using an electrospray ionization interface. Specifications on the used mass spectrometers and the parameter settings are provided in Supporting Information A6.

Thiacloprid was quantified in positive mode with the internal standard using TraceFinder 5.1 (Thermo Fisher Scientific Inc.) for peak integration. Additionally, a suspect screening using commonly known transformation products of thiacloprid was performed. Detailed information on quality control and quantification is provided in Supporting Information A7.

### Data Analysis

Total tissue bioconcentration factors (BCF_SS,total_ in L kg^–1^) under steady-state conditions were calculated for the toxicokinetic experiments II and III as the ratio of measured total tissue concentration at the end of the uptake phase (*C*_tissue,u_) and the average measured exposure concentration (*C*_water_)
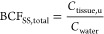
1

**Figure 3:**
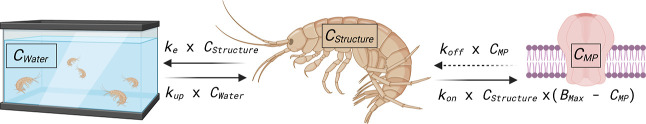
Illustration of the toxicokinetic-receptor model based on eqs 5 and 6. The volume of the water basin is assumed to be infinite compared to the volume of exposed gammarids. The weight ratio of the membrane protein (MP) and the structure compartment is defined as FMS (MP content, here 1%). Elimination from the MP compartment was set to zero (*k*_off_ = 0) because no elimination could be determined during the toxicokinetic experiments and model development.

Additionally, corresponding BCFs of the structure (further defined in the following section, Figure 3) compartment (BCF_SS,structure_ in L kg^–1^) for the concentration-dependent toxicokinetic experiment were determined by subtracting the elimination-resistant fraction at the end of the elimination phase (*C*_tissue,e_) from the concentration at the end of the uptake phase
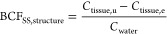
2

Receptor-binding properties of thiacloprid were modeled from the in vitro assay by determining the maximal irreversible binding parameter *B*_max_ (μmol kg_MP_^–1^) and the equilibrium dissociation constant *K*_d_ (nM). *B*_max_ is indicative of the maximum number of nAChR binding sites. Correspondingly, it is also a measure of the nAChR density of an organism if each receptor contains one specific binding site for thiacloprid. The equilibrium dissociation constant *K*_d_ represents the binding affinity of thiacloprid to nAChRs and is defined as the ligand concentration to achieve a half-maximum binding at equilibrium.^36^

The specific binding (*C*_specific_, μmol kg_MP_^–1^) model accounted for one-site, specific binding under equilibrium conditions
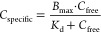
3where *C*_free_ (nM) is the concentration in the in vitro receptor-binding assay medium. The model was applied to the assay medium concentrations of 0 to 25 nM because unspecific binding at these concentrations was negligible compared to specific binding. Receptor-binding was modeled in GraphPad Prism 9.4 (GraphPad Software, Inc.). The modeled receptor-binding parameters were additionally confirmed using an unspecific binding model with an extended set of medium concentrations (Supporting Information A8).

Statistical analysis and data visualization were performed using GraphPad Prism 9.4 (GraphPad Software, Inc.). Significant differences between categorical variables were tested by ANOVA if not stated otherwise. The level of significance was set to 0.05. Normal distribution and homoscedasticity of the residuals were assumed. The law of error propagation was applied to all calculations.^37^

### Toxicokinetic Modeling

A toxicokinetic model, including receptor-binding (toxicokinetic-receptor model), was developed to describe the observed toxicokinetics of thiacloprid in *G. pulex*. Receptor models describing the kinetics of ligand–receptor complexes have been previously described for other organisms^38,39^ and discussed in the context of toxicokinetic–toxicodynamic models.^40^ Irreversible binding of thiacloprid to the nAChR was the core assumption of our model. Specifically, we assumed a two-compartment model with a structure (S) and a MP compartment incorporated into the structure compartment (Figure 3). While there is a bidirectional exchange between the structure compartment of the organism and the environment (i.e., water), the interaction of the structure compartment with the MP compartment is unidirectional due to irreversible receptor binding (*k*_off_ = 0). There is no direct interaction between water and the MP compartment. The model assumptions and reasoning are described in further detail in Supporting Information A9.

The formation of the ligand–receptor complex and its dissociation can be described through an ordinary differential equation. The change of the concentration of ligand-bound receptors, here approximated as the change of the thiacloprid concentration in the MP compartment *C*_MP_ (μmol kg_MP_^–1^) over time *t* (d), depends on the ligand (thiacloprid) concentration in the structure compartment *C*_structure_ (μmol kg_structure_^–1^) and the concentration of free receptors *N*_R_ (μmol kg_MP_^–1^) multiplied with the second-order rate *k*_on_ (kg_structure_ μmol^–1^ d^–1^). The first order rate *k*_off_ (d^–1^) determines the dissociation of the complex, depending on the concentration of ligand-bound receptors.
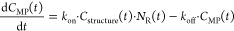
4

Our experimental results indicated an irreversible binding of thiacloprid at the temporal scale of the experiments. Furthermore, no parameter value for *k*_off_ significantly different from zero could be determined. Thus, the dissociation rate *k*_off_ was set to zero. With these observations, the assumption that the total number of receptors *N*_R0_ (μmol kg_MP_^–1^) is equal to the sum of ligand-bound receptors (*N*_RL_) and free receptors (*N*_R_ = *N*_R0_ – *N*_RL_), and further approximating *N*_R0_ as the maximal binding capacity *B*_max_ (μmol kg_MP_^–1^), eq 4 can be rearranged as follows
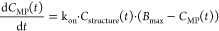
5

The concentration in the structure compartment *C*_structure_ can be determined using the following ordinary differential equation
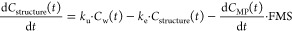
6where *k*_u_ (L kg_structure_^–1^ d^–1^) is the uptake rate, *C*_W_ (μM) is the water concentration of the exposure medium, *k*_e_ (d^–1^) is the elimination rate, and FMS (0.01 kg_MP_ kg_structure_^–1^) is the factor to correct the proportion of MP compared to the structure compartment.

The total tissue concentration *C*_total_ (μmol kg^–1^) can be approximated as
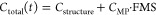
7

The modeled kinetic bioconcentration factor (BCF_kin,structure_ in L kg^–1^) in the structure compartment was determined as the ratio of *k*_u_ and *k*_e_
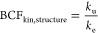
8

The internal concentrations derived from the toxicokinetic experiments were used to calibrate and validate the toxicokinetic-receptor model (eqs 5–7). Model calibration was done with data of constant exposures (0.05, 0.5, 5, 50, and 1500 μg L^–1^). The parameter space explorer^41^ was used for the optimization of the model parameter and to produce the confidence intervals of the model curves. The resulting best-fit model parameters were subsequently used to simulate model predictions. To validate this approach, the predictions were compared with both constant (5, 50, 500, and 5000 μg L^–1^) and pulsed (5 and 50 μg L^–1^) exposure scenarios. All calculations were performed in MATLAB 2021b using the Bring Your Own Model (BYOM) modeling platform (https://www.debtox.info/byom.html, version 6.2). Model scripts are provided on GitHub (https://github.com/NikaGoldring/TK-receptor).

## Results and Discussion

### Lipid and Protein Contents

The determined lipid contents (wet weight basis, mean ± SD, *n* = 3) were 0.7 ± 0.2% (toxicokinetic experiments, sampled in October and November) and 0.4 ± 0.2% (gammarids used for in vivo and in vitro receptor-binding assay, sampled in January). Lipid contents were in a similar range to that observed elsewhere.^32,42^ The observed decrease in lipid content from fall to winter may be due to the use of energy reserves, such as storage lipids.^42^ No lipid normalization of accumulated thiacloprid was performed, as lipid content was demonstrated to have no significant influence on the bioconcentration of polar organic contaminants in amphipods.^32^

The total protein content (wet weight basis) across the experiments ranged from 4.4 to 5.0% and is within the range reported for other gammarid populations.^42^ The average MP content was 1.0 ± 0.1%, which is higher than that reported for other aquatic invertebrates and methods.^35^

### Exposure Medium

The measured medium concentrations of all performed toxicokinetic experiments were within 20% of the nominal concentrations (Supporting Information B), which is in line with the OECD 305 requirements.^25^ Concentrations of the elimination medium were below the limit of quantification (generally 0.01 μg L^–1^, but 0.001 for the low exposure concentrations of 0.5 and 0.05 μg L^–1^), confirming a neglectable impact for reuptake from the medium, except for minor residues after exposure with concentrations ≥500 μg L^–1^. Relative O_2_ saturation (>85%), pH (7.9 ± 0.1) and water temperatures were stable (15.5 ± 1 °C) during the experiments.

### Exposure Concentration-Dependent Toxicokinetics

**Figure 4:**
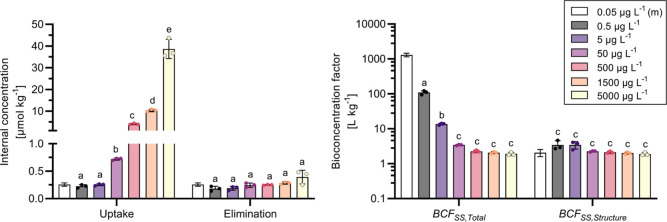
Internal concentrations (left) of thiacloprid at the end of the uptake and elimination phases at different exposure concentrations. Calculated uncorrected *BCF*_SS,total_ and the *BCF*_SS,structure_ (right). Data are presented as individual data points and mean ± SD (*n* = 3). Letters indicate significant differences between the groups (two-way ANOVA, *p* < 0.05, log transformation, Tukey’s post-hoc test). For 0.05 μg L^–1^ (m), data were modeled using the toxicokinetic-receptor model because no steady state was reached during this experiment (20 days).

The internal concentrations of gammarids exposed to different concentrations of thiacloprid (0.5–5000 μg L^–1^) increased significantly along the concentration gradient from an average of 0.23 ± 0.03 to 39 ± 4 μmol kg^–1^ at the end of the uptake phase (Figure 4). An approximation of steady state conditions was assumed based on the visually observed saturation in the kinetic experiments (i.e., pulsed exposure for ≥5 μg L^–1^) or kinetic measurements (0.5 μg L^–1^). The difference in internal concentration between the lowest and the highest concentration was only a factor of 150, despite a 10,000-fold difference in exposure concentration. The developed toxicokinetic model (Table 3) demonstrated that steady-state conditions would be reached within the exposure time window for all applied exposure concentrations except for 0.05 μg L^–1^. At the end of the elimination phase, no significant difference between remaining tissue concentrations was observed (0.18 ± 0.04 to 0.39 ± 0.13 μmol kg^–1^). Furthermore, no statistical difference between tissue concentrations at the end of the exposure and elimination phases was observed for concentrations equal to or lower than 5 μg L^–1^. Thus, the present data indicate a concentration dependence of the whole-body bioconcentration of thiacloprid in *G. pulex* due to a saturation of the second, elimination-resistant MP compartment.

In order to account for the two-compartment bioconcentration kinetics, two different BCFs were calculated (Figure 4). The BCF_SS,total_ was calculated from the tissue and medium concentration at the end of the uptake phase (eq 1), representing the whole body concentration. It showed a clear concentration dependence, ranging from 1.9 ± 0.2 L kg^–1^ at the highest exposure concentration toward a 60 times higher value (109 ± 14 L kg^–1^) at the lowest exposure concentration that reached a steady state (0.5 μg L^–1^). The BCF_SS,structure_, calculated by subtracting the elimination-resistant fraction from the total tissue concentration (eq 2), resulted in much more similar BCFs (2.5 ± 0.8 L kg^–1^), which were also in the range of the modeled BCF_kin,structure_ (2.0 L kg^–1^, eq 8) of the structure compartment. The difference between the BCF_SS,total_ and BCF_SS,structure_ was not significantly different for the high exposure concentrations (500 to 5000 μg L^–1^) due to the small relative contribution of the MP compartment to the whole body concentration.

All calculated BCF_SS,total_ in the present study were below the B criterion (BCF ≥ 2000 L kg^–1^)^25^ threshold and in range of those reported for neonicotinoids in *G. pulex* and other aquatic species (0.2–70).^20,28,43,44^ However, considering the increasing BCF_SS,total_ with decreasing exposure concentration—caused by the saturation of the MP compartment—the BCF of thiacloprid would continue to increase at lower, field-relevant concentrations. For instance, at concentrations below 30 ng L^–1^, the BCF_SS,total_ could increase above the regulatory threshold (BCF ≥ 2000 L kg^–1^) and at ≤12 ng L^–1^ above the very bioaccumulative criterion (BCF ≥ 5000 L kg^–1^), given a long enough exposure time. Measured neonicotinoid concentrations in surface waters are typically in this ng L^–1^ range.^5,16,22^ Thus, the concentration dependence of neonicotinoid accumulation would also explain the much higher accumulation of neonicotinoids observed in the field compared to laboratory studies.^22^

Previous investigations that determined internal concentrations of neonicotinoids in crustaceans^20^ may not have observed such two-compartment kinetics because of very high (mg L^–1^ range) exposure concentrations masking the elimination-resistant fraction (e.g., 5000 μg L^–1^, Figure 4). These high-exposure concentrations were applied due to the low acute toxicity of neonicotinoids toward crustaceans but probably also to guarantee a proper quantification of the compound in tissue samples. The methods applied in the present study facilitated the determination of medium and tissue concentrations of exposure experiments down to the ng L^–1^ range and allowed the detection of the elimination-resistant thiacloprid amount. Thus, testing bioconcentration at lower exposure concentrations may improve the detection and understanding of discrepancies between laboratory and field experiments.^22^

### Pulsed Exposure

**Figure 5:**
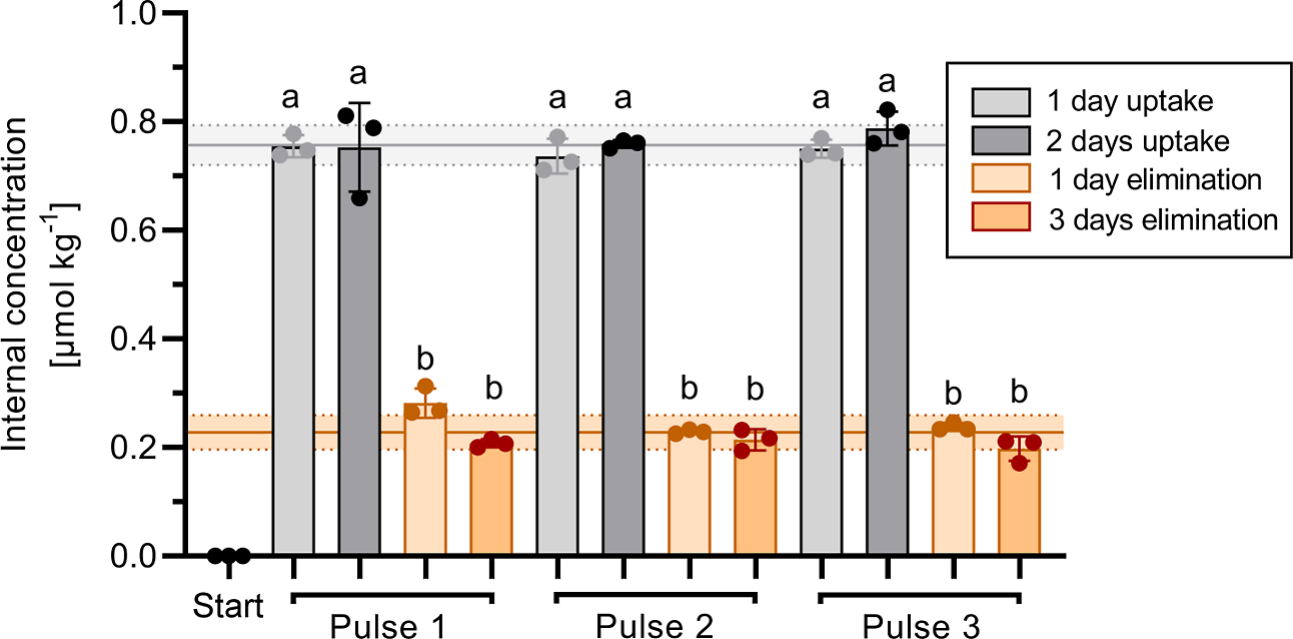
Internal thiacloprid concentrations in *G. pulex* sampled during the pulsed exposure experiment at 50 μg L^–1^. Lines represent the average (±SD, *n* = 18) tissue concentration determined in gammarids sampled during the uptake phase (gray) and elimination phase (orange). Significant differences within each exposure pulse concentration are indicated by letters (two-way ANOVA, *p* < 0.05, Tukey’s post-hoc test).

The measured tissue concentrations in gammarids exposed to three consecutive pulses of thiacloprid are shown in Figure 5. In the 50 μg L^–1^ exposure treatment, all internal concentrations were, on average, three times higher during the uptake phase after 1 and 2 days (0.76 ± 0.04 μmol kg^–1^) than during the elimination phase (0.23 ± 0.03 μmol kg^–1^). A similar pattern but lower internal concentrations at the end of the uptake phases were observed in the 5 μg L^–1^ treatment (Supporting Information A11). The measured internal concentrations, including the elimination-resistant fraction after 1 and 3 days of elimination, were similar to the concentrations measured in the concentration-dependent toxicokinetic experiments. Thus, the pulsed exposure experiment supported the observed elimination resistance of the MP compartment. Furthermore, it was demonstrated that the non-eliminating residues reached the maximum binding capacity already after the first exposure pulse and remained unchanged afterward. These results may help in interpreting toxic effects observed in pulsed exposure scenarios elsewhere.^19,45^

### Receptor-Binding Assays

**Figure 6:**
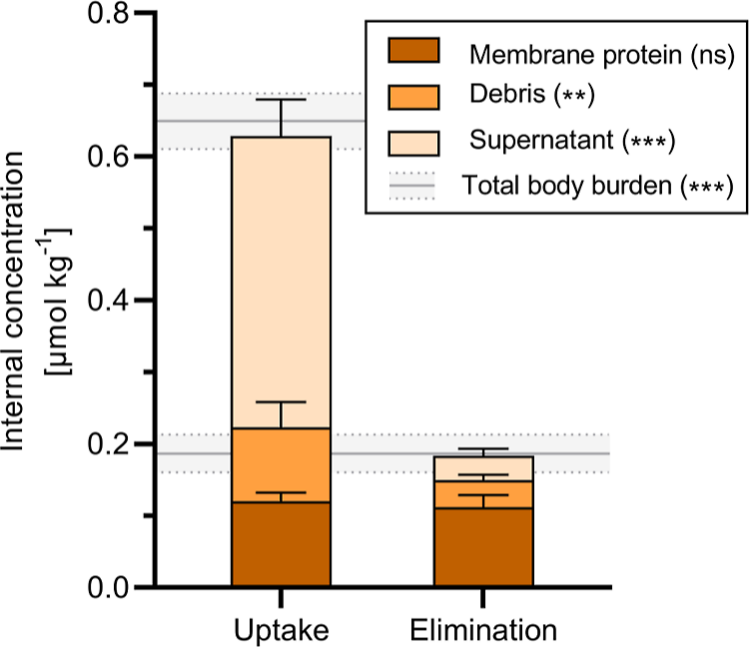
Thiacloprid concentrations recovered in different fractions of the in vivo nAChR binding assay presented as stacked bar plots. The gray line indicates the concentration determined from the unfractionated extract (lower line = elimination, upper line = uptake). Data are presented as mean ± SD (*n* = 6). Significant decreases from the end of uptake to the end of the elimination phase are indicated by asterisks (n.s. = *p* ≥ 0.05, ** = *p* < 0.01, *** = *p* < 0.001; two-way ANOVA, Tukey’s post-hoc test).

The amount of thiacloprid recovered in the in vivo receptor-binding assay matched the concentrations of the conventionally extracted (whole body burden) samples of both the exposure and elimination phases (Figure 6). After the uptake phase, most thiacloprid was recovered in the supernatant (64%), followed by MPs (19%) and debris (16%). Thiacloprid associated with different fractions may be interpreted as follows: (1) supernatant = free thiacloprid (i.e., structure compartment) or thiacloprid detached from MPs or associated with MPs that were not separated during ultracentrifugation, (2) debris = thiacloprid that was not extracted by the DM (i.e., incomplete MP extraction, association with the exoskeleton, incorporation into un-lysed tissue), and (3) membrane protein = thiacloprid associated with membrane proteins such as the nAChRs. Binding or sorption to the exoskeleton (i.e., debris fraction, exuviae analysis Supporting Information A4) seemed to be of low importance for thiacloprid body burdens, other than what was suggested for other chemicals in crustaceans in earlier reports.^20,22^

The amount of thiacloprid in the supernatant and the debris decreased significantly by more than 90 and 60%, respectively, during the elimination phase. In contrast, thiacloprid concentrations in MP remained constant from the exposure to the elimination phase. At the end of the elimination phase, thiacloprid associated with MP accounted for the largest amount (61%) of the total body burden. The in vivo receptor-binding assay indicated that the elimination-resistant fraction of thiacloprid in *G. pulex* may be caused by irreversible binding to parts of the MPs, such as the nAChRs.

Parameters from the one-site specific binding model (*R*^2^ = 0.83, Supporting Information A8) of the in vitro ligand binding assay are presented in Table 2. Similar parameters were estimated with the unspecific binding model (*B*_max_ = 5.5 and *K*_d_ = 0.41, Supporting Information A8). While the CI of *B*_max_ was narrow (<15%), *K*_d_ was estimated with considerable uncertainty due to the insufficient coverage of the binding isotherm in the proximate region of *K*_d_ (Scatchard plot). However, even in previous studies covering lower exposure concentrations and with a more sensitive radio-labeled method, similar confidence intervals were obtained.^35^ An extrapolation of *B*_max_ to the whole organism (*C*_*B*_max__), correcting for MP recovery after the in vitro assay (24%) and MP content (1%) resulted in a whole-body concentration of 0.24 μmol kg^–1^. Such concentration is in a similar range to the elimination-resistant fraction determined in the toxicokinetic experiments, including the estimated *C*_*B*_max__ of the toxicokinetic-receptor model (Table 3) and an in vivo binding assay. Thus, the utilization of in vivo and in vitro receptor-binding assays seemed to be sufficient to upscale receptor-binding processes to the whole organism level, which provides potential for novel toxicokinetic research approaches.

**Table 2:** Thiacloprid-Binding Parameters Estimated with the One-Site Specific Binding Model^a^

parameter	best fit	95% CI	unit	explanation
*B*_max_	5.7	5.1–6.4	μmol kg_MP_^–1^	maximal binding capacity (MP)
*K*_d_	0.41	0.15–0.85	nM	equilibrium dissociation constant
*C*_*B*_max__	0.24	0.21–0.27	μmol kg^–1^	*B*max scaled to organism level

a Parameters are presented with the corresponding 95% CIs (confidence intervals). *R*^2^ = 0.83. *C*_*B*_max__ = maximal nAChR-bound thiacloprid extrapolated to the whole body of *G. pulex* predicted using the MP content FMS (1%) and the MP recovery rate of 24% throughout the in vitro assay. *C*_*B*_max__ is equal to the receptor density in the whole organism, assuming one binding site per receptor.

**Table 3:** Optimized Model Parameters of the Toxicokinetic Receptor Model with Their Corresponding 95% CIs, *R*^2^ = 0.99^a^

parameter	best fit	95% CI	unit	Explanation
*k*_u_	10.6	8.5–13.6	L kg_structure_^–1^ d^–1^	uptake rate
*k*_e_	5.2	4.2–6.7	d^–1^	elimination rate
*k*_on_	200	37.6–200^b^	Kg_structure_ μmol^–1^ d^–1^	association rate for the ligand–receptor complex
*k*_off_	0	fixed	d^–1^	dissociation for the ligand–receptor complex
*B*_max_	25.0	22.7–29.4	μmol kg_MP_^–1^	maximal binding capacity (MP)
*C*_*B*_max__	0.25	0.23–0.29	μmol kg^–1^	*B*_max_ scaled to organism level

a *C*_*B*_max__ = Maximal nAChR-bound thiacloprid amount extrapolated to the whole body of*G. pulex*using the membrane protein content FMS (1%).

b Boundary of the parameter space explorer.

The *B*_max_ values obtained from the in vivo receptor-binding assay may be compared to existing studies with imidacloprid, as both imidacloprid and thiacloprid were suggested to bind to the same nAChR-binding site (one of five subunits) in cockroach neurons.^46^ Therefore, the *B*_max_ values of the two neonicotinoids may also be indicative for the receptor densities in different arthropod species. Maloney et al.^35^ reported *B*_max_ values for imidacloprid in 13 invertebrate species ranging from 51 × 10^–6^ to 6.5 μmol kg_MP_^–1^. The *B*_max_ for thiacloprid in *G. pulex* was closest to the values reported for imidacloprid in *Chironomus riparius* and *C. dilutus* larvae.^35^ Since nAChRs are not only located in the peripheral and the central nervous systems but also in muscular tissues,^47^ the high binding capacity in both organism types may result from a high proportion of muscular tissue (Supporting Information A12). A compartmentation of the neonicotinoid imidacloprid into the nervous system and muscle tissue was also indicated in a radio-imaging study on gammarids.^148^ Furthermore, it should be noted that similar molar concentrations of imidacloprid at the end of the elimination phase from toxicokinetic experiments with two other *G. pulex* populations^31,48^ were comparable to the observations for thiacloprid in the present study. These findings support the suggested similar nAChR-binding capabilities of the two neonicotinoids in gammarids.

The methods from Maloney et al.^35^ using radiolabeled imidacloprid could be sufficiently adapted to less specialized laboratory equipment and the measurement of unlabeled ligands. However, the adaptation resulted in several drawbacks, such as the need for larger sample volumes and time-consuming ultracentrifugation. Possible optimizations, such as the reduction of the assay to a microplate layout to reduce centrifugation steps and losses, are discussed in Supporting Information A13.

### Toxicokinetic-Receptor Model

**Figure 7:**
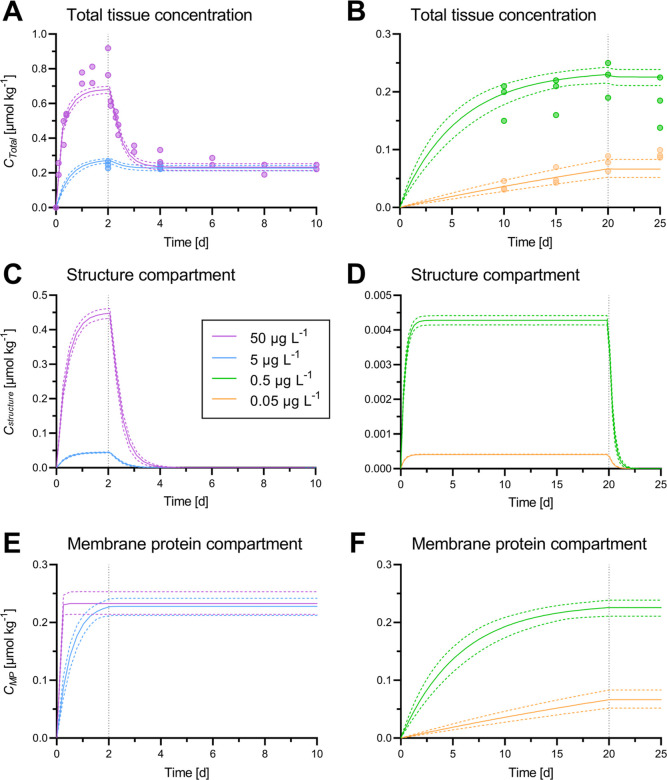
Total thiacloprid tissue concentrations (A,D), structure compartment (B,E), and MP compartment (C,F) concentrations presented as measured values (dots) and toxicokinetic-receptor model fits (lines) from the model calibration. Colored dotted lines represent the 95% CIs. Gray dotted lines indicate the change from the uptake to the elimination phase. Please note the different *y*-axis scales. The presented MP concentration is up-scaled to the total tissue concentration to allow a better comparison. Underlying model parameters are provided in Table 2. The complete dataset is plotted in Figure S6.

The thiacloprid concentrations in *G. pulex* tissue (Figure 7) showed a steady increase during the exposure phase of the kinetic experiments. The increase in tissue concentrations was slowing down considerably between days 1 and 2 at exposure concentrations ≥5 μg L^–1^. During the first day of the elimination phase, thiacloprid was rapidly removed from the structure compartment, but no elimination occurred from the MP compartment, which determines the remaining total tissue concentration. This behavior is consistent with the observations of the receptor-binding assays.

The determined model parameters are provided in Table 3. The calibrated best-fit model parameter resulted in an overall *R*^2^ of 0.99 in describing the measured total internal concentration of thiacloprid in *G. pulex*. The quality of the fit by visual examination was deemed satisfactory (Figures 7 and S6). The profile likelihoods (Figure S7, plots on the diagonal) of *k*_u_, *k*_e_, and *B*_max_ were well defined (i.e., u-shaped and crossing the critical value on both ends). *k*_on_ could not be identified, as this process seemed to be much faster than *k*_u_ and the time resolution of the observations. Therefore, the optimization algorithm hit the arbitrarily set upper limit for this parameter. Thus, the association process might be seen as instantaneous, given the available dataset. The likelihood-based joint-confidence regions (Figure S7, scatter plots) showed a strong correlation of the model parameters *k*_u_ and *k*_e_, expressed in their narrow-shaped ellipse. The calibrated model showed high accuracy in predicting the measured concentrations of the validation datasets for both constant (Figure S8) and pulsed exposure scenarios (Figure S9). Physiological inactivity of gammarids (Supporting Information A11) had a noticeable impact by reducing toxicokinetic rates but only minor effects on *B*_max_ and *BCF*_kin,structure_.

One assumption of the developed model was irreversible binding, which resulted in a fixed value of zero for *k*_off_. No elimination of thiacloprid during the present experiments could be observed by statistical or modeling means. However, this assumption may not hold true for much longer experimental times. For instance, a recovery time of 45 days was determined by toxicokinetic–toxicodynamic (TK–TD) modeling for imidacloprid-exposed daphnids,^20^ but no recovery was identified for gammarids in a different study.^48^ In competition-binding assays, it was demonstrated that the binding affinity of neonicotinoids is much higher than that of acetylcholine, but neonicotinoids may still be removed if acetylcholine is available in large excess.^49^ This mechanism may eventually result in a slow recovery of affected nAChRs. Furthermore, organisms may recover by deconstructing affected receptors and generating new receptors dynamically. These mechanisms may also lead to lower *B*_max_ values at very low exposure concentrations (≪ 0.5 μg L^–1^), which take a considerably longer time to reach the maximal *C*_MP_ concentration.

The developed model assumed well-mixed compartments as a simplification. However, this might not be appropriate for the representation of receptors in different organs. It is known that different nAChRs of various organ types (i.e., muscles and nervous system) are built from different subunits.^50^ The proportion of these receptor/subunit types may change across species, seasons, and developmental states, thus affecting the suitability of the applied MP content normalization. Furthermore, this may limit the extrapolation of the total nAChR density based on receptor-bound neonicotinoids.

The profile likelihood analysis revealed an identification problem with the parameter *k*_on_, whose upper boundary could not be distinguished from infinity (Figure S7, plots on the diagonal). This problem can be associated with so-called “fast kinetics” extensively discussed by experts before.^51^ That is, when fast kinetics are observed, the steady-state is reached before the first measurement. In the present study, this was the case for the receptor-bound fraction because no independent kinetic measurements of the receptor-bound fraction were feasible. Thus, the data do not hold the exact information about how quickly a steady state between the structure and the MP compartment is achieved. To minimize the effect of this parameter and avoid numerical problems on the joint-confidence regions, the fixed boundary of the parameter space explorer (i.e., 200) was used as an upper boundary of *k*_on_. Consequently, also the assumption of instantaneous binding could be another way of simplifying the present model. The described observation is in line with the high receptor affinity of thiacloprid to nAChRs, indicating that the speed of in vivo receptor-binding kinetics are limited by the initial uptake of thiacloprid into the structure compartment.

Biotransformation of thiacloprid in amphipods was neither reported in the literature nor found in a screening for reported biotransformation products (e.g., thiacloprid amide) in bacteria^52^ (Supporting Information A7). Thus, in order to also display toxicokinetics of neonicotinoids or other receptor-bound compounds that are biotransformed in amphipods (e.g. imidacloprid^43,53^), the presented model may be extended by considering biotransformation. However, further research would be needed in order to understand and implement the exact mechanisms of biotransformation, such as compartment-dependent biotransformation and binding of biotransformation products to the nAChRs.

### Considerations for Risk Assessment

In the present study, we demonstrated that irreversible binding to MPs such as the nAChRs explains the observed elimination resistance^22,31,32^ of neonicotinoids from amphipod tissue. Consequently, this elimination-resistant fraction may explain the delayed toxic effects and irreversible damages toward aquatic arthropods that were previously reported.^17,18,45,53^ In fact, the MP-associated fraction may be interpreted as either irreversible damage to the nAChR or continuous exposure due to elimination resistance, depending on the point of view. The here-provided mechanistic insights may help to improve the understanding of toxicokinetics, toxicodynamics, and adverse outcome pathways of neonicotinoids in arthropods. Such considerations may be important for the risk assessment of neonicotinoids, as well as their replacement candidates (i.e., flupyradifurone^9^) and other contaminants with (irreversible) receptor-binding properties. Furthermore, existing TK–TD modeling approaches^19,48,53,54^ may be updated based on the present findings.

The standard bioaccumulation assessment, according to OECD 305^25^ using fish, generally assumes one-compartment kinetics, an independence of bioaccumulation parameters from exposure concentrations and a relevance of bioaccumulation only for compounds with high log *K*_OW_ values. However, our toxicokinetic investigations on neonicotinoids in aquatic invertebrates demonstrated a strong exposure concentration dependence due to a maximum binding capacity and no elimination from the MP compartment. These mechanisms may result in BCFs above the threshold value for the B criterion (2000) at concentrations typically observed in the environment (ng L^–1^ range).^5,16^ In contrast to multi-compartment kinetics caused by sorption to exoskeleton/cuticula of aquatic invertebrates,^55^ the here-reported second compartment consists of a bioactive, and thus toxicologically relevant, fraction. Similar mechanisms for elimination-resistant bioactive fractions may exist for other compound classes with observed multi-compartment kinetics, such as strobilurins in amphipods.^26,32^ However, further research is needed to understand the underlying mechanisms and their toxicological relevance. In order to account for concentration-dependent bioaccumulation, a category such as “elimination-resistant” or “receptor-bound” may be important for establishing new testing guidelines, i.e., for the proposed bioaccumulation studies using arthropods.^27,56^ With our developed toxicokinetic-receptor model, we provide the required modeling platform for such implementations.

Furthermore, the usefulness of environmental threshold concentrations for elimination-resistant compounds such as neonicotinoids may be reconsidered.^16^ Exposed organisms may accumulate neonicotinoids over their lifetime and eventually reach saturation of the nAChRs, regardless of the exposure concentration. Additionally, this resistance toward elimination may explain trophic magnification in arthropods and transfer from aquatic to terrestrial food webs observed for neonicotinoids elsewhere.^57^ Furthermore, environmental parameters may have an impact on the toxicokinetics of neonicotinoids. For instance, temperature was demonstrated to exert an exponential relationship with uptake and elimination rates in amphipods.^32^ This may result in much faster saturation of the nAChRs, especially if high water concentrations co-occur with higher temperatures, such as during a run-off event in the summer, and consequently enhance the exposure risks toward aquatic arthropods. However, further investigations of the interaction of temperature and neonicotinoid exposure are needed to evaluate this risk.

## Supporting Information Available

Data on the measured concentrations of kinetic experiments, concentration-dependent toxicokinetic experiments, pulsed exposure experiments, in vivo receptor-binding experiments, and in vitro receptor-binding experiments (XLSX)
Neonicotinoid insecticides, test animal acclimation, lipid content, investigations on exoskeleton sorption, protein content, online SPE LC-HRMS/MS settings and quality control, in vitro receptor-binding assay, details on the toxicokinetic-receptor model, impact of physiological activity on toxicokinetics, pulsed exposure, gammarid cross-section, and possible receptor-binding assay optimizations (PDF)

## Abbreviations

ACh: acteylcholine
APW: artificial pond water
BCF: bioconcentration factor
TK: toxicokinetic
TK–TD: toxicokinetic–toxicodynamic
MP: membrane protein
nAChR: nicotinic acetylcholine receptor
