# Supplementary material for: Elimination Resistance: Characterizing Multi-compartment Toxicokinetics of the Neonicotinoid Thiacloprid in the Amphipod Gammarus pulex Using Bioconcentration and Receptor-Binding Assays
Source: Environ Sci Technol. 2023 Jun 7;57(24):8890–901. doi: 10.1021/acs.est.3c01891 (PMC10286312; doi:10.1021/acs.est.3c01891)
Supplement: Supplementary file 2 — es3c01891_si_002.pdf [file es3c01891_si_002.pdf]

Supporting Information A:

Elimination resistant: Characterizing multi-compartment toxicokinetics of the neonicotinoid thiacloprid in the amphipod *Gammarus pulex* using bioconcentration and receptor binding assays

Johannes Raths[1,2], Linda Schinz[1,2], Annika Mangold-Döring[3], Juliane Hollender[1,2]*

[1]Department of Environmental Chemistry, Swiss Federal Institute of Aquatic Science and Technology - Eawag, Überlandstrasse 133, 8600 Dübendorf, Switzerland

[2]Institute of Biogeochemistry and Pollutant Dynamics, ETH Zürich, Universitätstrasse 16, 8092 Zürich, Switzerland

[3]Department of Aquatic Ecology and Water Quality Management, Wageningen University, P.O. Box 47, 6700 Wageningen, The Netherlands

*Corresponding author: juliane.hollender@eawag.ch

Includes 21 pages, 12 figures and 12 tables

Table of Contents

SI A1: Neonicotinoid insecticides S3

SI A2: Test animal acclimation S3

SI A3: Lipid content S4

SI A4: Investigations on exoskeleton sorption S5

Exuviae collection S5

Exoskeleton rinsing S5

SI A5: Protein content S7

SI A6: Online SPE LC-HRMS/MS settings S8

SI A7: Online SPE LC-HRMS/MS quality control S11

SI A8: *In vitro* receptor binding assay – specific and unspecific binding model S12

SI A9: Details on the toxicokinetic-receptor model S13

Assumptions S13

Symbols, parameters and equations S14

Model calibration S15

Model validation S17

SI A10: Impact of physiological activity on toxicokinetics S18

SI A11: Pulsed exposure S19

SI A12: Gammarid cross section S20

SI A13: Possible receptor binding assay optimizations S20

References S21

List of Tables

Table S1: Relative amount of thiacloprid associated with exuviae of G. pulex. S5
Table S2: Schedule of the online-SPE on the QExactive and the QExactive Plus mass spectrometers. S9
Table S3: Schedule of the online-SPE on the Exploris 240 mass spectrometer. S9
Table S4: Gradient of the liquid chromatography. S10
Table S5: Source parameters used for HRMS/MS measurement with the QExactive. S10
Table S6: Source parameters used for HRMS/MS measurement with the QExactive Plus. S10
Table S7: Source parameters used for HRMS/MS measurement with the Orbitrap Exploris............. S10
Table S8: MS parameters used for HRMS/MS measurements. R S11
Table S9: Quality control parameters of LC-ESI-HRMS/MS measurements....................................... S11
Table S10: Thiacloprid receptor binding parameters.. S13
Table S11: Parameter symbols and explanations for the TK-receptor model. S14
Table S12: Comparison of the toxicokinetic parameters modelled for alive and dead gammarids ... S19

List of Figures

Figure S1: Molecular structures of neonicotinoid insecticides. S3
Figure S2: Internal thiacloprid concentrations in alive and dead gammarids...................................... S6
Figure S3: Illustration of the total protein and membrane protein extraction method. S7
Figure S4: Saturation curve of the in vitro receptor binding assay. S12
Figure S5: One-site, non-specific binding model of thiacloprid. S12
Figure S6: Model calibration fits. S15
Figure S7: Parameter space plot, calibration. S16
Figure S8: Model validation for constant exposure scenarios. S17
Figure S9: Model validation for pulsed exposure scenarios. S17
Figure S10: Measured and modelled thiacloprid concentrations in dead gammarids. S18
Figure S11: Internal thiacloprid concentrations in *G. pulex* sampled during the pulsed exposure experiment at 5 µg L^{-1} exposure concentration. S19
Figure S12: Sagittal cross section of a cryoembedded specimen of *G. pulex*. S20

SI A1: Neonicotinoid insecticides

An overview of the seven commercially available neonicotinoid insecticides and nicotine is presented in Figure S1.

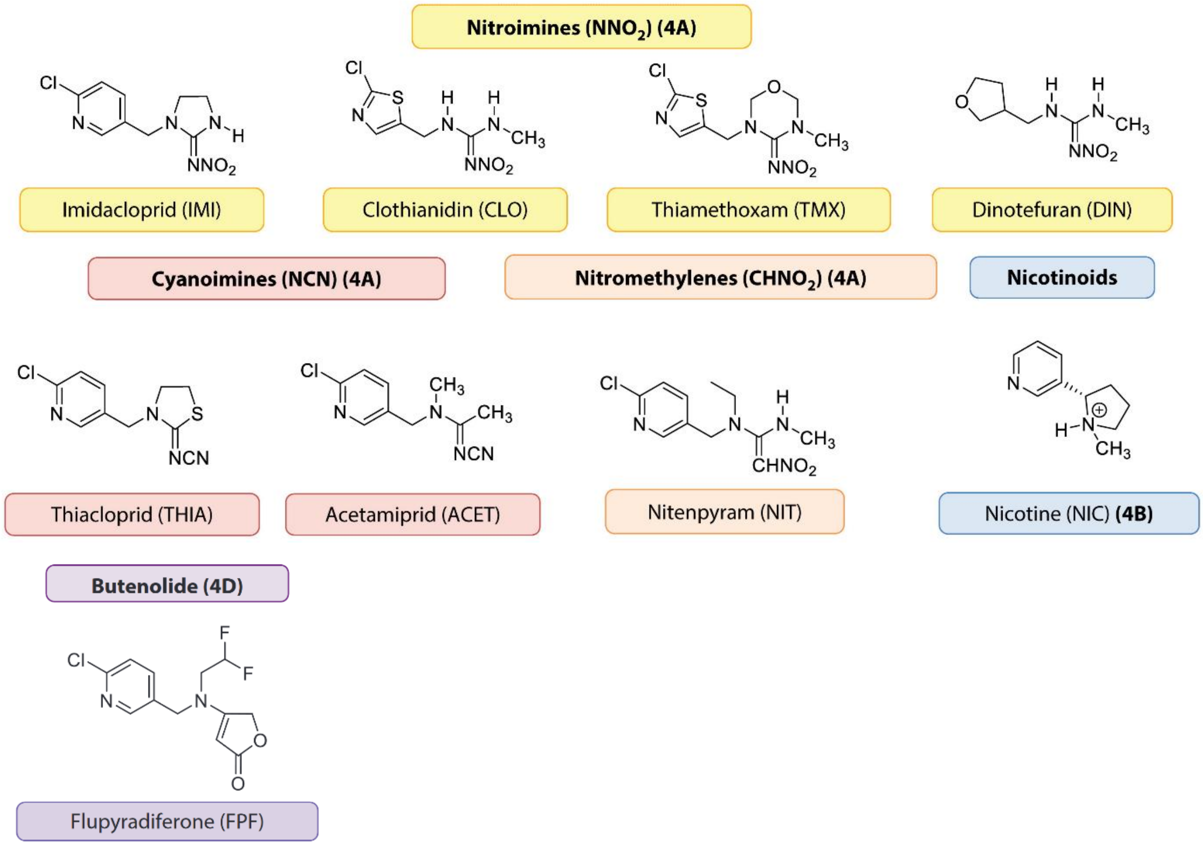

Figure S1: Molecular structures of neonicotinoid insecticides, the replacement candidate flupyradifurone and nicotine. Thiacloprid is highlighted in red. Adapted from Casida (2018).[1]

SI A2: Test animal acclimation

After transport to Eawag facilities, the gammarids were transferred to glass aquaria filled with river water from the sampling site. The aquaria were aerated and placed into an incubator programmed to 12:12 h light-dark cycles. During the acclimation period prior to the start of the experiments (≥ 3 d), the water in the aquaria was progressively replaced to 100 % (v/v) artificial pond water (APW) (pH = 7.9 ± 0.3, prepared according to Naylor et al.[2]). In addition, the temperature in the incubator was increased gradually to 15.5 ± 1°C by the end of acclimation. The amphipods were fed ad libitum with the leaves collected at the sampling site. Juvenile, pregnant or animals visibly infected with acanthocephalan parasites were sorted out and not introduced into any experiment.

SI A3: Lipid content

The lipid content of gammarids was determined gravimetrically according to Raths et al.[3]. Samples were taken from the gammarid batch of experiments I to III (3 replicates, 4 gammarids each), and the batch for experiments V and VI (3 replicates, 4 gammarids each) shortly before (< 1 h) the corresponding experiments started. Prior to the lipid extraction, 1.5 mL glass vials were labelled incubated (90 °C, 2 h) and then weighed. Next, snap frozen gammarids were sampled into 2 mL Eppendorf vials and weighed. 300 mg of silica beads and 1 mL cyclohexane/isopropanol 5:4 (v/v) were added and samples homogenized in two runs (6 m s^{-1}, 15 s, FastPrep-24TM, MPITM). Next, 0.6 mL of nanopure water (NPW) were added, samples were vortexed (30 s) and centrifuged (1000 x g, 10 min, 4 °C). Then, the organic supernatant was transferred into the 1.5 mL glass vials using glass pasteur pipettes. Subsequently, 0.55 mL of cyclohexane/isopropanol 87:13 (v/v) were added to the residues and samples were vortexed (30 s) and centrifuged (1000 x g, 10 min, 4 °C) again. Then, the supernatant was once again removed and combined with the previous extract. The extraction solvent was then evaporated at 60 °C overnight and vials were weighed the next morning. Finally, the gammarid lipid content was calculated as the ratio of the lipid weight to the gammarid wet weight.

SI A4: Investigations on exoskeleton sorption

Previous research on gammarids and daphnids suggested sorption to the crustacean exoskeleton as a possible artefact in bioconcentration experiments.[4,5] Thus, the objective of this experiment was to determine the amount of thiacloprid bound to the exoskeletons of *G. pulex*. As muscular tissue of gammarids is very closely grown together with the exoskeleton, no sufficient dissectioning methods could be established. Instead, exuviae from moulted gammarids were collected as described earlier[5]. Additionally, experiments where thiacloprid-exposed gammarids were washed with different detergents were performed.

Exuviae collection

Exuviae from moulted gammarids were collected during the kinetic experiment (TK kinetic experiment I, 50 μg L^{-1}). The exuviae were sampled, extracted, and analysed in the same manner as other gammarid samples.

During the uptake phase, the shedded exoskeleton accounted for 1.7 to 2.4 % (m/m) of thiacloprid compared to the total body burden at the same time point (Table S1). During the elimination phase, the contribution increased to 6.6–17 %. It should be noted that the number of exuviae sampled was small, especially during the elimination phase (n = 1) leading to high uncertainty for the upscaling of the results. However, the elimination resistant fraction accounted for approximately one third of the total tissue concentration at the end of the uptake phase and would be assumed to account for the major proportion of the total tissue concentration at the end of the elimination phase. Thus, the exoskeleton bound fraction would not explain the elimination resistant residues observed during the toxicokinetic experiments and our findings were different from observations reported for daphnids.[4] Higher proportions of exoskeleton associated fractions (i.e. up to 24%) were reported by Miller et al.[5] for ionised pharmaceuticals where this mechanism may be of more relevance.

Table S1: Relative amount of thiacloprid associated with exuviae of G. pulex.

Phase	Time [h]	Replicates	Exuviae associated fraction (%)
**Uptake**	24	6	2.2
	34	3	2.4
	48	2	1.7
**Elimination**	51	1	6.6
	72	1	17
	96	1	7.9

Exoskeleton rinsing

In this experiment, gammarids (collected in October, together with experiment II and III) were exposed to 50 µg L^{-1} thiacloprid for two days and then transferred to uncontaminated medium for another two days. The amphipods were sampled in triplicates after two and four days, respectively. This experiment was performed with live and dead (heat shock-euthanised) gammarids. Each treatment was tested in a separate aquarium. Only the tank containing live gammarids was aerated and received leaf disks during the elimination phase. In contrast to the standard method, at the end of the experiment the amphipods were removed from the aquaria, rinsed with one of three solutions, and subsequently rinsed with nanopure water. Thereby, it was tested whether the detergents were able to remove thiacloprid potentially sorbed to the exoskeleton of *G. pulex*. The solutions were the detergent triton X-100, dimethyl sulfoxide (DMSO) and a commercially available soap (MayaEasy Soft). DMSO is a solvent often used to accelerate the dissolution of chemicals in toxicology and pharmacology.[6] Triton X-100, on the other hand, is a widely used surfactant for lysing cells and to make cells permeable.[7] The triton X-100 and DMSO solutions were prepared in 0.1% dilution in NPW. The soap detergent was produced by adding 1 drop of soap to 0.5 L of NPW. As a control treatment, gammarids were washed

twice with NPW. Afterwards, the gammarids were homogenized and extracted with methanol as described in the manuscript.

The internal concentrations in live gammarids at the end of the uptake (48 h) and elimination phase (96 h) rinsed with different detergents are presented in Figure S2. The internal concentrations of alive and dead gammarids washed with DMSO, triton X-100 and soap did not differ significantly from the control gammarids washed with NPW or each other (one-way ANOVA, $p \geq 0.05$). All measured tissue concentrations were in the range of previous toxicokinetic experiments. A larger variance of data for dead gammarids was observed and may be explained by disintegration caused by microbial growth and slower kinetics in lifeless tissue (i.e. because of no sterilisation).

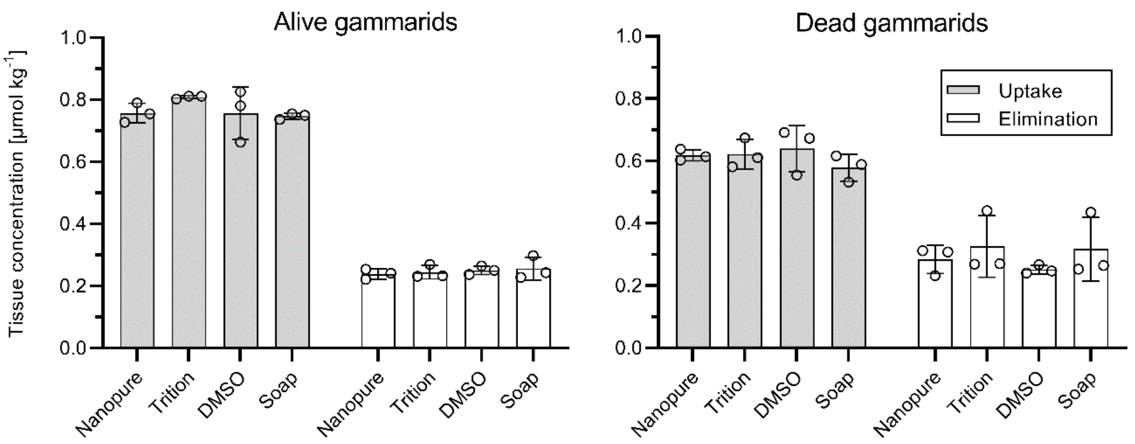

Figure S2: Internal thiacloprid concentrations (mean ± SD, n = 3) in alive (left) and dead (right) gammarids washed with NPW, and triton X-100, DMSO and soap after 48 h of exposure (grey) and after 48 h of elimination (white). Individual data points are presented as dots. No significant influence of washing solution was detected (one-way ANOVA, p ≥ 0.05).

The detergent rinsing experiment demonstrated that the elimination resistant fraction could not be washed of the gammarids using any of the present solutions. Because some of the detergents are specifically used to remove organic contaminants sorbed to the surface of a sample it was concluded that sorption to the exoskeleton of amphipods would not explain the non-eliminating thiacloprid fraction.

SI A5: Protein content

The protein contents were determined in gammarids from the batch of experiments I to III (18 gammarids), and the batch of experiments V and VI (49 gammarids). Samples were analysed in sets of approximately 200 mg gammarid per vial (= 1 mL DM per 200 mg ww).

The complete (membrane) protein extraction workflow is illustrated in Figure S3. The total protein concentration was determined from the combined extracts SN1 and SN2. The present, adapted approach using a cooled pre-cooled tissue lyser recovered an approximately 20% higher total protein content compared to homogenization using an automatic pestle motor as described by Maloney et al.[8]

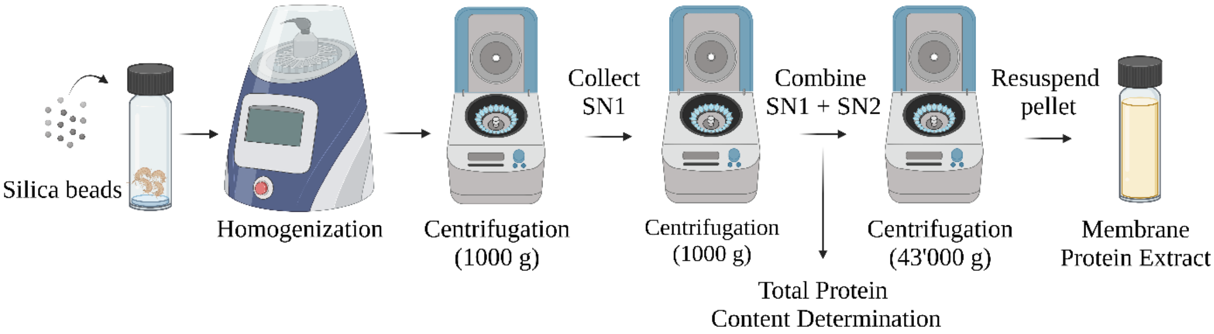

Figure S3: Illustration of the total protein and membrane protein extraction method. SN1 and SN2 designate the supernatants after the first and the second centrifugation run at 1000 x g.

Detailed methods on the Pierce BCA Protein Assay Kit (ThermoFisher Scientific):

In short, 200 µL of the Working Reagent were added to wells of a 96-well plate. Afterwards, 25 µL of protein suspension or calibration standard were added to the wells. The calibration standards were produced by diluting bovine serum albumin (BSA) with dissociation medium as described in the BCA Protein Assay Kit manual. All standards and samples were prepared in duplicates. After adding standards and samples to the 96-well plate, the covered well plate (in a plastic bag to avoid evaporation) was incubated (30 min, 37 °C). The plate was cooled down to room temperature and read within 10 min after incubation using a plate reader (Tecan InfiniteR 200 PRO). Protein concentrations were then determined with a linear standard calibration curve. The R^2 of the calibration curve of all assays was ≥ 0.98. Protein suspensions were stored at -20 °C until performance of the ligand binding assays.

The membrane protein content and the protein content in the supernatant (F_{Prot}, in % wet weight) were calculated as the protein weight normalised by the gammarid wet weight:

$$F_{Prot} = \frac{c_{Prot} \cdot V_{Extract}}{W_{Gammarid}} \quad \text{(Eq. S1)}$$

where C_{Prot} (mg L^{-1}) is the protein concentration determined in the BCA assay, $V_{Extract}$ (L) the extraction volume (4 mL for membrane proteins and 7 mL for proteins remaining in the supernatant) and $W_{Gammarid}$ (mg) the gammarid wet weight (ww). The total protein is the sum of the membrane protein and the supernatant protein content.

In a direct comparison, the optimised protein extraction methods adapted from Maloney et al.[8] provided a 50% higher total protein yield (3.8 ± 0.2% ww versus 2.5 ± 0.2% ww, mean ± SD, n = 2) than by our previous methods[3] which were based on Janssen et al.[9].

SI A6: Online SPE LC-HRMS/MS settings

Thiacloprid concentrations were determined on three different mass spectrometers (Thermo Fisher Scientific Inc.), which are the QExactive (kinetic experiment I, concentration dependence II and III), QExactive Plus (*in vivo* and *in vitro* nAChR binding assays V and VI) and Exploris 240 (exoskeleton rinsing, pulsed exposure IV).

On the QExactive, thiacloprid was measured in polarity switching mode. On the QExactive Plus and the Exploris 240, samples were measured in positive ionisation mode. Additional information on the online SPE and LC-ESI-HRMS/MS settings are provided in Table S2 to Table S8. Peaks were integrated and concentrations were determined with the software TraceFinder 5.1 (Thermo Fisher Scientific Inc.), using a linear standard calibration and the THI/ISTD area ratio. Calibrants were weighted by the inverse of their quantity in the least squares optimisation. The R^2 of the calibration curves of all measurements was ≥ 0.99.

To prepare the online SPE column, 8-9 mg of Oasis HLB (15 µm particle size, Waters) were added to an empty stainless steel SPE cartridge (20 mm x 2.1 mm, BGB Analytik AG). Next, the cartridge was filled with about 8-9 mg of a mix of anion exchanger Strata X-AW, cation exchanger Strata X-CW (both ion exchangers: 30 µm, Phenomenex, UK) and Env+ (70 µm, Biotage, Sweden) in a ratio of 1:1:1.5 (X-AW : X-CW : Env+).

For the measurement on the QExactive and QExactive Plus, 20 mL of NPW and 100 µL of sample extracts were added to 20 mL glass vials. For the measurement on the Exploris, 100 µL of the samples were added to 10 mL of NPW.

Table S2: Schedule of the online-SPE on the QExactive and the QExactive Plus mass spectrometers.

Time [min]	Acetonitrile [µL min^{-1}]	Ammonium acetate solution (2 mM) [µL min^{-1}]	SPE step
**0**		200	Elution of the sample from the cartridge (with elution pump) and washing of the loop.
**0.1**	4000		
**1.1**	4000		
**1.2**		4000	
**6.7**		4000	
**6.8**		400	
**7.3**		400	
**7.4**	400		Loading of the new sample into the loop and conditioning of the cartridge.
**12.5**	400		
**12.6**		400	
**18.4**		400	
**18.5**		1270	Enrichment of the new sample.
**32.1**		1270	
**34.5**		1270	
**34.7**		1270	
**35**		200	

Table S3: Schedule of the online-SPE on the Exploris 240 mass spectrometer.

Time [min]	Acetonitrile [µL min^{-1}]	Ammonium acetate solution (2 mM) [µL min^{-1}]	SPE step
**0**		200	Elution of the sample from the cartridge (with elution pump) and washing of the loop.
**0.1**	2000		
**1.1**	2000		
**1.2**		2000	
**6.7**		2000	
**6.8**		400	
**7.3**		400	
**7.4**	400		Loading of the new sample into the loop and conditioning of the cartridge.
**12.5**	400		
**12.6**		400	
**18.4**		400	
**18.5**		635	Enrichment of the new sample.
**32.1**		635	
**34.5**		635	
**34.7**		200	
**35**		200	

Table S4: Gradient of the liquid chromatography. Water and methanol were both acidified with 0.1% (vol.) formic acid. Chromatographic separation was performed with a reversed-phase column (Atlantis T3 C18 column, 5 µm, 3x150 mm, Waters, Batch No 0151351351)

Time [min]	H_2O [µL min^{-1}]	MeOH [µL min^{-1}]
**0.0**	0.260	0.040
**5.0**	0.260	0.040
**20.0**	0.015	0.285
**29.0**	0.015	0.285
**29.5**	0.260	0.040
**35.0**	0.260	0.040

*Table S5: Source parameters used for HRMS/MS measurement with the QExactive (Thermo Fisher Scientific Inc.) mass spectrometer. *External mass calibration with an amino acid solution (11 amino acids with m/z between 116 and 997) in positive and negative ionisation mode.*

Parameter	Value
**Sheath gas (nitrogen) flow rate**	40 L min^{-1}
**Auxiliary gas (nitrogen) flow rate**	15 L min^{-1}
**Capillary temperature**	350 °C
**S-lens RF level**	50
**Mass calibration**	External*
**Spray voltage**	4 kV (positive ionisation mode)
	3 kV (negative ionisation mode)

*Table S6: Source parameters used for HRMS/MS measurement with the QExactive Plus (Thermo Fisher Scientific Inc.) mass spectrometer. *External mass calibration with an acid solution (11 amino acids with m/z between 116 and 997) in positive and negative ionisation mode.*

Parameter	Value
**Sheath gas (nitrogen) flow rate**	40 L min^{-1}
**Auxiliary gas (nitrogen) flow rate**	15 L min^{-1}
**Capillary temperature**	320 °C
**S-lens RF level**	50
**Mass calibration**	External*
**Spray voltage**	4 kV (positive ionisation mode)

*Table S7: Source parameters used for HRMS/MS measurement with the Orbitrap Exploris 240 (Thermo Fisher Scientific Inc.) mass spectrometer. *External mass calibration with an acid solution (11 amino acids with m/z between 116 and 997) in positive and negative ionisation mode.*

Parameter	Value
**Sheath gas (nitrogen) flow rate**	40 L min^{-1}
**Auxiliary gas (nitrogen) flow rate**	10 L min^{-1}
**Capillary temperature**	320 °C
**Mass calibration**	External*
**Spray voltage**	3.4 kV (positive ionisation mode)

Table S8: MS parameters used for HRMS/MS measurements. Resolution presented at m/z 200. MS2 isolation window: 1 m/z.

Mass spectrometer	Parameter	Value
**QExactive**	Resolution MS1	70k
	Scan range	100-1000 m/z
	Polarity	Switching mode
	Resolution MS2	17.5k
**QExactive Plus**	Resolution MS1	70k
	Scan range	150-2000 m/z
	Polarity	Positive
	Resolution MS2	30k
**Exploris**	Resolution MS1	140k
	Scan range	100-1000 m/z
	Polarity	Positive
	Resolution MS2	17.5k

SI A7: Online SPE LC-HRMS/MS quality control

Quality control parameters of the online SPE LC-HRMS/MS performance are presented in Table S9. The high matrix factors in the receptor binding assays are likely to be caused by phosphate salts, which originate from the dissociation medium, and were not completely removed in the online-SPE process. Limits of quantification were not limiting any of the gammarid extract and medium analysis.

Table S9: Quality control parameters of LC-ESI-HRMS/MS measurements: R^2 of the calibration fit, relative recovery (RR), limit of detection (LOD_{Matrix}) and limit of quantification (LOQ_{Matrix}) in gammarid extracts, medium samples or for membrane protein extraction (MP).

Instrument	R^2	Sample type	RR[a] [%]	Matrix factor	LOD_{Matrix} [µg kg^{-1}]	LOQ_{Matrix} [µg kg^{-1}]
**QExactive**	≥ 0.99	Gammarid	109	0.29	0.84	1.68
		Medium	101	0.95	0.05	0.11
**QExactive Plus**	≥ 0.99	Gammarid		0.58	0.06	0.06
		Medium		0.71	0.01	0.01[c]
		MP[b]		3.35	0.001	0.001
**Exploris 240**	≥ 0.99	Gammarid		0.25	0.10	0.20
		Medium		1.15	0.004	0.01

[a]Relative recovery was determined once on the Q Exactive.
[b]Values averaged for debris, supernatant and membrane protein extracts from the receptor binding assays.
[c]The LOQ was 0.001 µg L^{-1} for the TK experiment II, due to a higher SPE injection volume.

A suspect screening on biotransformation products of thiacloprid was performed based on reported biotransformation products in bacteria[10]. For this purpose, the by full scan acquired HRMS/MS data were screened for the exact masses $[M+H]^+$ of the reported biotransformation products. Additionally, a reference standard of the main biotransformation product thiacloprid amide was analysed in the calibration and relative recovery samples. No precursor ion of any biotransformation product was detected in gammarid samples, but thiacloprid amide was found in the calibration and recovery samples as expected.

SI A8: ***In vitro*** **receptor binding assay – specific and unspecific binding model**

The saturation curve of the specific binding model at concentrations of 1 to 25 nM (n = 4) is presented in Figure S4.

For an unspecific binding model, additional receptor binding assays with concentrations of 50, 100 and 250 nM were performed (n = 4, 2 and 2).

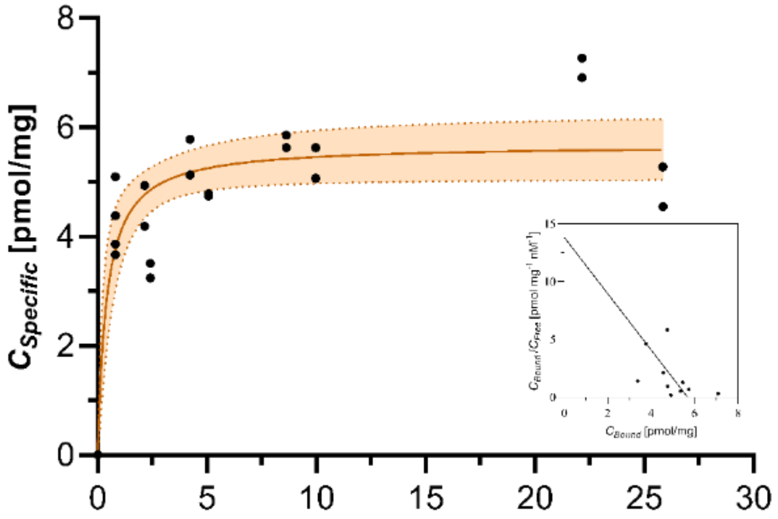

Figure S4: Saturation curve of the in vitro receptor binding assay with thiacloprid and G. pulex membrane protein. Measured data are presented as dots. The non-linear regression of the one-site, specific binding model and the 95 % CIs are presented as orange line and shaded area, respectively. The corresponding Scatchard plot is shown in the lower right corner. pmol mg^{-1} equals µmol kg^{-1}.

The unspecific binding model for one specific binding site under equilibrium conditions was modelled by adding the slope of the nonspecific binding *NS* (pmol mg^{-1} nM^{-1}):

$$C_{Bound} = \frac{B_{Max} \cdot C_{Free}}{K_d + C_{Free}} + NS \cdot C_{Free} \qquad \text{(Eq. S2)}$$

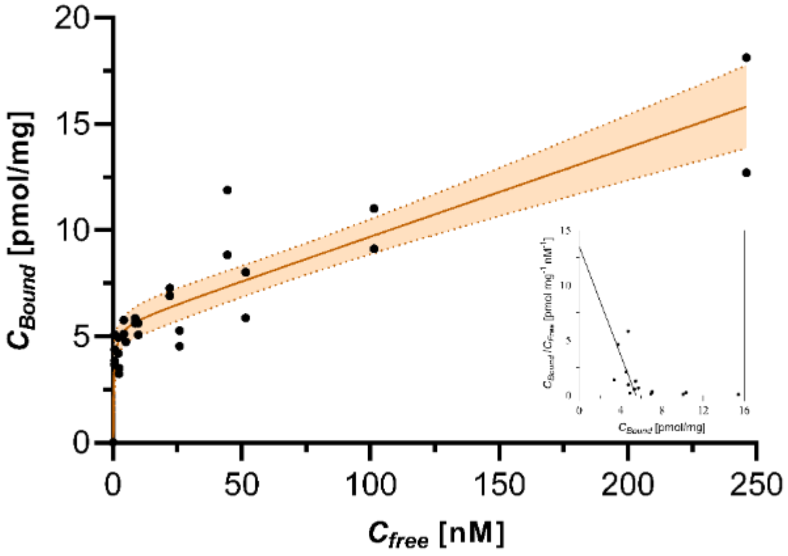

Figure S5: One-site, non-specific binding model of thiacloprid bound to membrane proteins of G. pulex. Measured receptor binding assay data are presented as dots. The non-linear regression and the 95 % CIs are presented as orange line and shaded area, respectively. The corresponding Scatchard plot is shown in the lower right corner. pmol mg^{-1} equals µmol kg^{-1}.

The corresponding unspecific binding model and the calculated parameters are presented in Figure S5 and Table S10, respectively. The estimates of B_{Max} and K_d were very similar (< 4% deviation) than the values calculated from the specific binding model.

Table S10: Thiacloprid receptor binding parameters estimated with the one-site, unspecific binding model. Parameters are presented with the corresponding 95% CIs. R^2 = 0.87. C_{BMax} = maximal nAChR bound thiacloprid extrapolated to the whole body of G. pulex using a membrane protein recovery rate of 24.3% across the in vitro assays.

Parameter	Best fit	95% CI	Unit	Explanation
B_{Max}	5.5	3.7 – 7.3	µmol kg_{MP}^{-1} or pmol mg_{MP}^{-1}	Maximal binding capacity (membrane protein)
K_d	0.41	-0.04 – 1.77	nM	Equilibrium dissociation constant
NS	0.04	0.03 – 0.05	µmol kg_{MP}^{-1} nM^{-1} pmol mg_{MP}^{-1} nM^{-1}	Slope of the nonspecific binding
C_{BMax}	0.24	0.15 – 0.30	µmol kg^{-1}	B_{Max} scaled to organism level

SI A9: Details on the toxicokinetic-receptor model

Assumptions

The *in vivo* and *in vitro* assays of this study showed evidence of an elimination resistance predominantly present in the membrane protein fraction. Moreover, the nACh receptors, as membrane proteins, are the molecular target of thiacloprid.[11] Due to this evidence, the underlying assumptions of this model are the following:

1) The organism is represented as a two-compartment system with a structure compartment representing the debris and supernatant fraction of the *in vivo* assay, and a membrane protein compartment representing the fraction of the membrane protein, including receptors. Both compartments are homogeneous (well-mixed) compartments. The external water (i.e., environment) compartment is also well-mixed, infinitely large, and not influenced by the organism.
2) The uptake flux of the chemical from the water into the structure compartment is proportional to the external water concentration, and the uptake flux of the chemical from the structure to the membrane protein compartment is proportional to the concentration in the structure compartment as well as the concentration within the membrane protein compartment (saturation effect).
3) The elimination flux of the chemical from the structure compartment to the environment is proportional to the concentration of the structure compartment. There is no elimination of the chemical from the membrane protein compartment.
4) The measured chemical concentrations in the membrane protein compartment approximate the concentration of ligand-bound receptors in the organism.

Symbols, parameters and equations

A summary of all model parameters and state variables used in the model development, including the best fit parameters of the calibration, is provided in Table S11.

*Table S11: Parameter symbols and explanations for the TK-receptor model. Calibrated best-fit values are for the exposure of Gammarus pulex with thiacloprid. *Boundary of the parameter space explorer*

Symbol	Explanation	Unit	Best fit	95% CI
C_w	Exposure concentration	µM	forcing, exposure scenario	
C_{Total}	Total tissue concentration	µmol kg^{-1}		
FMS	Factor to normalize for membrane protein content assuming a density of 1 for both membrane protein and structure	Kg$_{MP}$ kg$_{Structure}^{-1}$	Fixed value	
State variables				
$C_{Structure}$	Concentration of ligand in structure compartment	µmol kg$_{Structure}^{-1}$	state variable	
C_{MP}	Concentration of ligand in membrane protein compartment	µmol kg$_{MP}^{-1}$	state variable	
Parameters				
k_u	Uptake rate	L kg$_{Structure}^{-1}$ d^{-1}	10.6	8.5 – 13.6
k_e	Elimination rate	d^{-1}	5.2	4.28 – 6.7
k_{on}	Association rate for ligand-receptor complex	Kg$_{Struccture}$ µmol^{-1} d^{-1}	199.6	37.6 – 200*
k_{off}	Rate for dissociation of ligand-receptor complex	d^{-1}	fixed to 0	
B_{max}	Maximal binding capacity	µmol kg$_{MP}^{-1}$	25.00	22.7 – 29.4
R^2	Coefficient of determination	-	0.99	

The final model equations are summarized in Eq. S3 to S5.

$$\frac{dC_{MP}(t)}{dt} = k_{on} \cdot C_{Structure}(t) \cdot \left(B_{max} - C_{MP}(t)\right) \quad \text{(Eq. S3)}$$

$$\frac{dC_{Structure}(t)}{dt} = k_u \cdot C_w(t) - k_e \cdot C_S(t) - \frac{dC_{MP}(t)}{dt} \cdot FMS \quad \text{(Eq. S4)}$$

$$C_{tot} = dC_{Structure} + C_{MP} \cdot FMS \quad \text{(Eq. S5)}$$

Model calibration

The plots of the model calibration are presented in Figure S6 (tissue concentrations) and Figure S7 (parameter space).

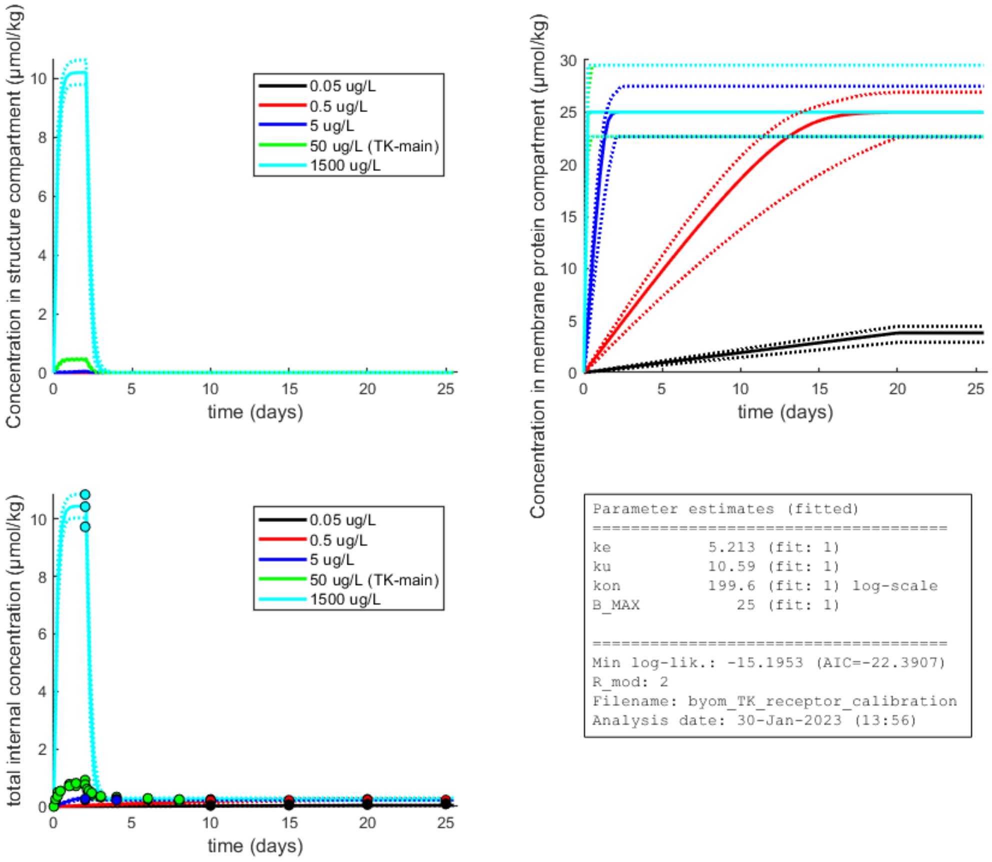

Figure S6: Model calibration. Concentrations of thiacloprid in the structure and membrane protein compartment, as well as the combined total internal concentration in Gammarus pulex over time are plotted. Solid lines show the modeled concentrations for the respective exposure concentrations (i.e., 0.05, 0.5, 5, 50 , and 1500 μg·L^{-1}) and dotted lines represent their lower and upper confidence intervals. The measured total body concentrations are show as dots. Fit: 1 indicates that a parameter was fitted.

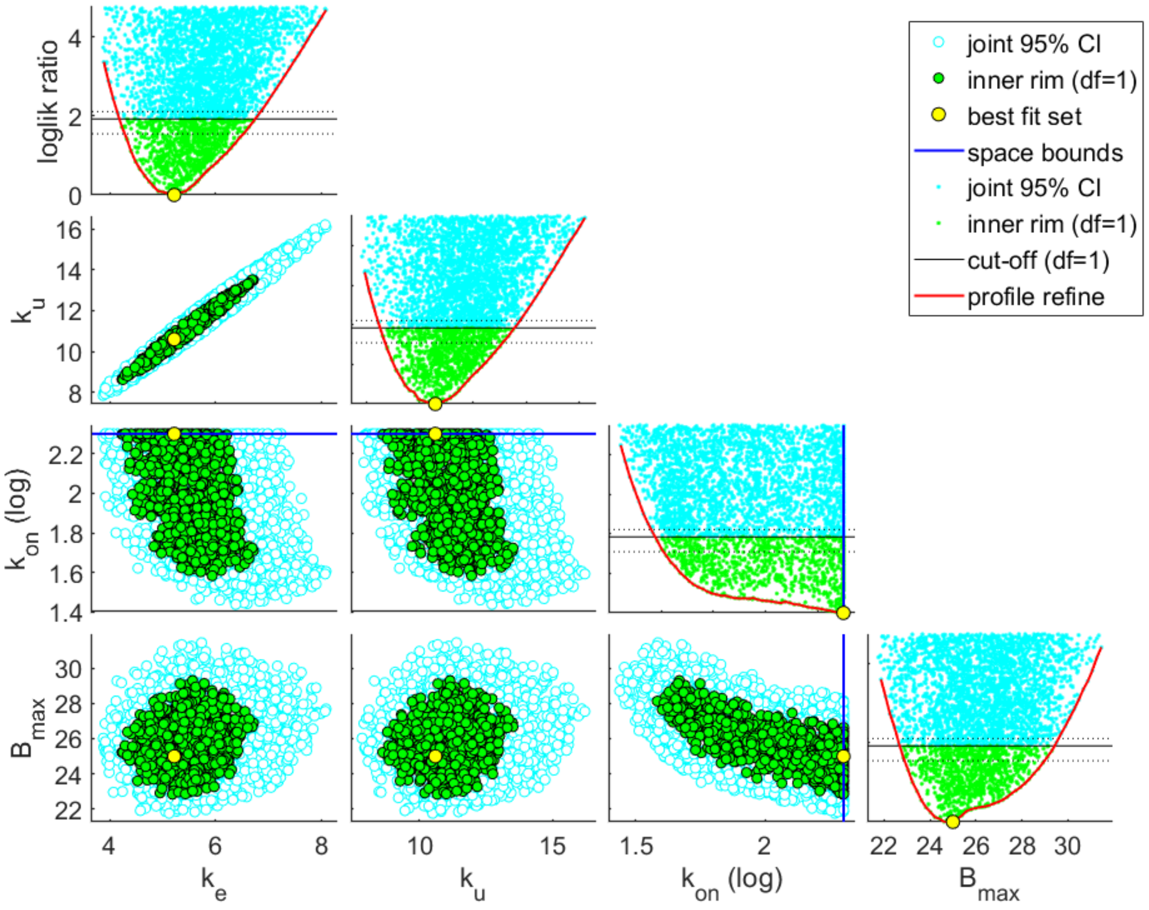

Figure S7: Parameter space plot, calibration. The u-shaped plots on the diagonal show the profile likelihoods for the individual parameters. The scatter plots underneath are the 95% joint confidence regions. The yellow dots mark the best-fit values for each parameter and green dots show the parameter sets within the critical value (i.e., under the horizontal black line). The parameter symbols and units can be obtained from Table S11.

Model validation

The plots of the model validation are presented in Figure S8 and Figure S9.

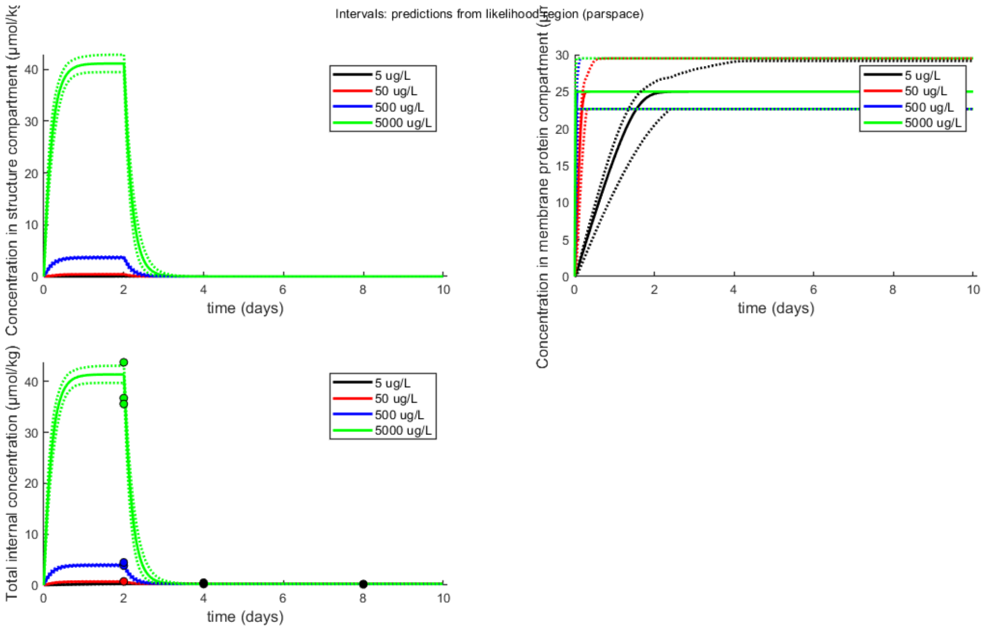

Figure S8: Model validation for constant exposure scenarios. Concentrations of thiacloprid in the structure and membrane protein compartment, as well as the combined total internal concentration in Gammarus pulex over time are plotted. Solid lines show the simulated concentrations for the respective scenario concentrations (i.e., 5, 50 , 500 and 5000 µg·L^{-1}) and dotted lines represent their lower and upper confidence intervals. The measured total body concentrations are show as dots.

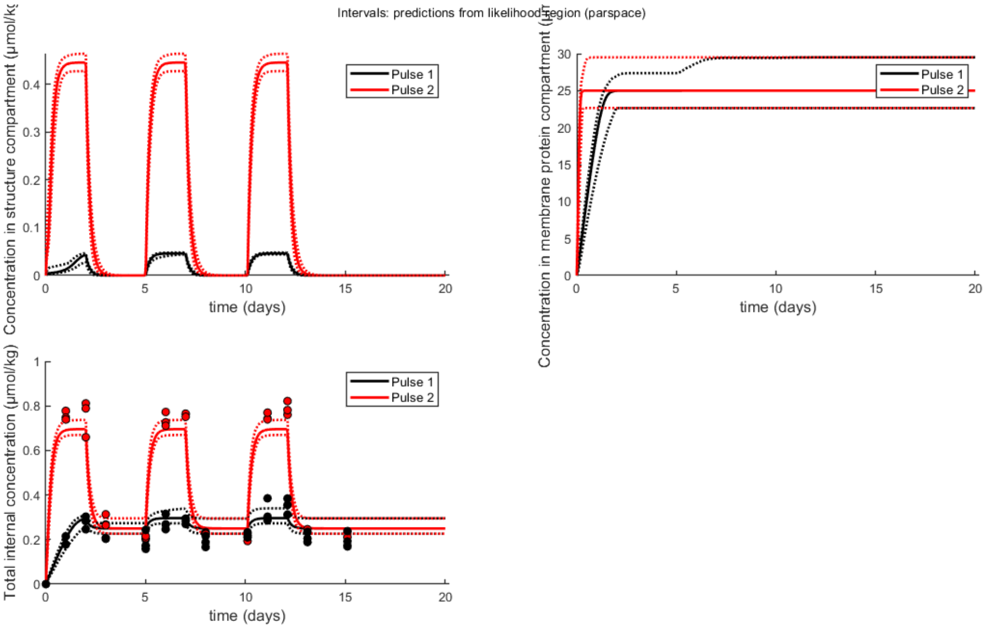

Figure S9: Model validation for pulsed exposure scenarios. Concentrations of thiacloprid in the structure and membrane protein compartment, as well as the combined total internal concentration in Gammarus pulex over time are plotted. Solid lines show the simulated concentrations for the respective pulse scenario concentrations (i.e., 5 and 50 µg·L^{-1}) and dotted lines represent their lower and upper confidence intervals. The measured total body concentrations are show as dots.

SI A10: Impact of physiological activity on toxicokinetics

To differentiate between active (i.e. filtration, enzyme activity) and passive (i.e. diffusion) toxicokinetic processes, an additional setup, analogous to toxicokinetic experiment I, was conducted with dead gammarids. Gammarids were heat shock inactivated (few seconds at 53 °C) and exposed in a separate aquarium. Aeration and feeding were omitted to reduce bacterial growth that promotes disintegration of the dead gammarids. Due to disintegration, the elimination phase lasted only 6 days. For toxicokinetic receptor modelling the parameter k_{on} was fixed to 200, as the dataset would not allow a proper estimation of the parameter.

The measured and modelled concentrations in dead gammarids are presented in Figure S10. At the end of the uptake phase, the total tissue concentration in dead gammarids was with 0.72 ± 0.03 µmol kg^{-1} slightly lower compared to 0.84 ± 0.11 µmol kg^{-1} in alive gammarids. At the end of the elimination phase, the tissue concentrations in dead gammarids were 0.30 ± 0.02 µmol kg^{-1} compared to 0.23 ± 0.02 in alive gammarids. The measured concentrations were similar to experiments for investigations on exoskeleton sorption (SI A4, exoskeleton rinsing) for both alive and dead gammarids. Despite slower kinetic rates, both the steady state concentration as well as the non-eliminating fraction of accumulate thiacloprid were on very similar levels between both alive and dead gammarids. The higher residual thiacloprid concentration in dead gammarids at the end of the elimination phase could potentially be caused by disintegration of the dead gammarids, leading to a lower sample weight (average reduction of 37% after two days of elimination).

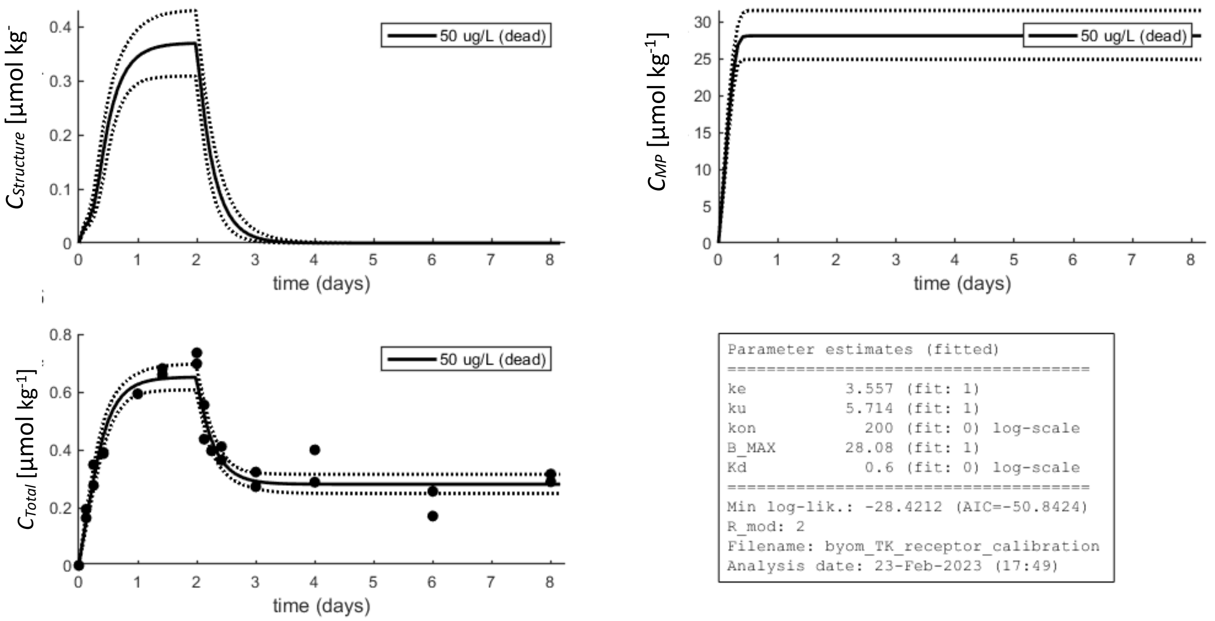

Figure S10: Measured (dots) and modelled (lines) thiacloprid concentrations in different compartments of dead gammarids using the toxicokinetic-receptor model. 95% CIs are indicated by dotted lines. Underlying toxicokinetic parameters are presented in the lower right corner and compared to alive gammarids in Table S12.

The toxicokinetic parameters are compared with the parameters for alive gammarids in Table S12. The present experiment showed that a loss of physiological activity (as is the case in dead gammarids) had a considerable impact on the kinetic rates (strongest on k_u, - 50%) but much smaller effect (-20%) on the $BCF_{kin,Structure}$ or the elimination resistant fraction C_{BMax}. A similar effect of physiological activity (i.e. filtration) on toxicokinetics was indicated earlier[3,12,13]. The heat shock inactivation of gammarids has shown to inhibit biotransformation enzymes[3] and would imply that also membrane proteins, such as nAChRs, denature. However, Saitoh et al.[14] reported that it took approximately 6 min to thermally destabilize 50% of nAChRs at 60 °C, which is a much longer heat exposure than in the present study

(few seconds). Thus, it appears likely that the nAChR in dead gammarids were still mostly intact and irreversible binding explains the elimination resistant thiacloprid fraction also in dead gammarids.

Table S12: Comparison of the toxicokinetic parameters modelled for alive and dead gammarids exposed to thiacloprid. k_{on} was set to 200 for dead gammarids.

State	k_u [L kg^{-1} d^{-1}]	k_e [d^{-1}]	B_{Max} [µmol$_{MP}$ kg^{-1}]	C_{Bmax} [µmol kg^{-1}]	$BCF_{kin,Structure}$ [L kg^{-1}]	R^2
Alive	10.6 (8.5–13.6)	5.2 (4.2 – 6.7)	25.0 (22.7 – 29.4)	0.25 (0.23 – 0.29)	2.0	0.99
Dead	5.7 (4.9–6.7)	3.6 (2.8 – 4.5)	28.1 (24.9 – 31.4)	0.28 (0.25 – 0.31)	1.6	0.97

SI A11: Pulsed exposure

In the 5 µg L^{-1} treatment (Figure S11), internal concentrations were only significantly higher after the third pulse compared to elimination phase samples (and 24 h exposure of the first pulse). The internal concentrations were slightly higher during the uptake phase (0.28 ± 0.06 µmol kg^{-1}, n = 18) than during the elimination phase (0.20 ± 0.03 µmol kg^{-1}).

The observed pattern provides a more blurry vison on the pattern of the 50 µg L^{-1} pulsed exposure scenario. The low concentration after 24 h of the first pulse may be caused by the initial uptake (k_u) into gammarid tissue limiting the transfer of thiacloprid from the exposure medium towards the membrane protein, as explained with the toxicokinetic-receptor model. The estimated time to steady state at 5 µg L^{-1} exposure is about 3 to 4 days. This may explain the slight increase in uptake phase concentration across the three pulses.

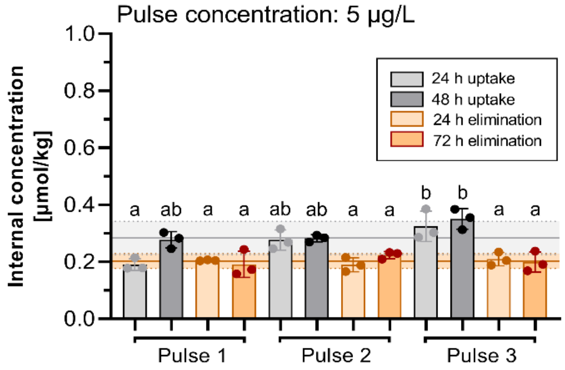

Figure S11: Internal thiacloprid concentrations in G. pulex sampled during the pulsed exposure experiment at 5 µg L^{-1} exposure concentration. Lines represent the average (± SD, n = 18) tissue concentration determined in gammarids sampled during the uptake phase (grey) and elimination phase (orange). Significant differences within each exposure pulse concentration are indicated by letters (two-way ANOVA, $p < 0.05$, Tukey's post-hoc test).

SI A12: Gammarid cross section

A gammarid sagittal cross section of *G. pulex* indicating the high proportion of muscle tissue is presented in Figure S12.

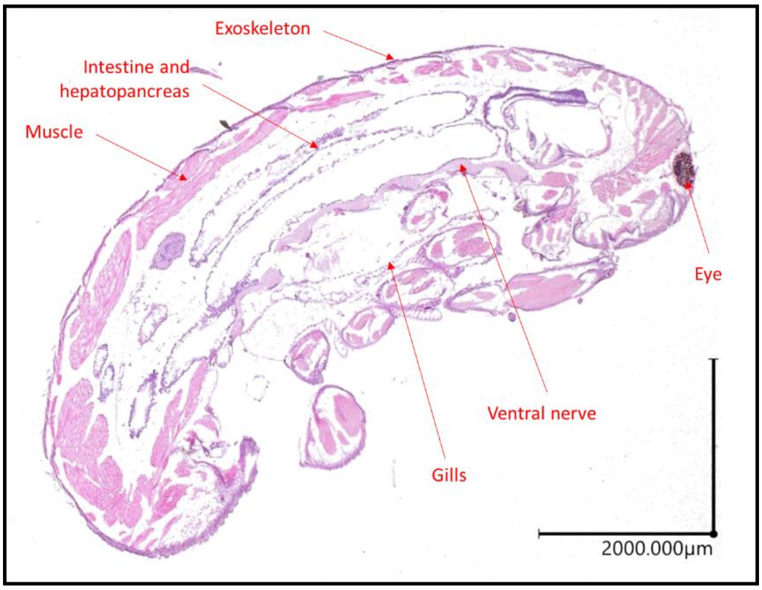

Figure S12: Sagittal cross section (16 µm) of a cryoembedded (2.5% carboxymethyl cellulose) specimen of G. pulex. Hematoxylin-eosin stain.

SI A13: Possible receptor binding assay optimizations

Even though the sensitivity acquired by online-SPE LC-HRMS/MS measurements was sufficient for determination of the thiacloprid concentrations in the assay, time and resource requirements exceed the ones of the radio ligand assay. However, the present method would allow to distinguish between multiple ligands (i.e. competition assays) and potential transformation products. Furthermore, centrifugation was much more time consuming compared to filtration resulting in much lower sample throughput, higher workload, and potentially higher membrane protein loss. Due to the higher volumes required for ultracentrifugation, higher initial amounts of membrane protein were needed in order to obtain reasonable ratios of membrane protein and assay medium. The reduction of the assay to a microplate layout would minimise most of the drawbacks of the present method and should be considered for future applications for non-radioactively labelled compounds.

References

(1) Casida, J. E. Neonicotinoids and Other Insect Nicotinic Receptor Competitive Modulators: Progress and Prospects. *Annu. Rev. Entomol.* **2018**, *63* (1), 125–144. https://doi.org/10.1146/annurev-ento-020117-043042.

(2) Naylor, C.; Maltby, L.; Calow, P. Scope for Growth in Gammarus Pulex, a Freshwater Benthic Detritivore. *Hydrobiologia* **1989**, *188* (1), 517–523. https://doi.org/10.1007/BF00027819.

(3) Raths, J.; Švara, V.; Lauper, B.; Fu, Q.; Hollender, J. Speed It up: How Temperature Drives Toxicokinetics of Organic Contaminants in Freshwater Amphipods. *Glob. Change Biol.* **2023**, *29* (5), 1390–1406. https://doi.org/10.1111/gcb.16542.

(4) Li, H.; Zhang, Q.; Su, H.; You, J.; Wang, W.-X. High Tolerance and Delayed Responses of Daphnia Magna to Neonicotinoid Insecticide Imidacloprid: Toxicokinetic and Toxicodynamic Modeling. *Environ. Sci. Technol.* **2021**, *55* (1), 458–467. https://doi.org/10.1021/acs.est.0c05664.

(5) Miller, T. H.; McEneff, G. L.; Stott, L. C.; Owen, S. F.; Bury, N. R.; Barron, L. P. Assessing the Reliability of Uptake and Elimination Kinetics Modelling Approaches for Estimating Bioconcentration Factors in the Freshwater Invertebrate, Gammarus Pulex. *Sci. Total Environ.* **2016**, *547*, 396–404. https://doi.org/10.1016/j.scitotenv.2015.12.145.

(6) Christou, M.; Kavaliauskis, A.; Ropstad, E.; Fraser, T. W. K. DMSO Effects Larval Zebrafish (Danio Rerio) Behavior, with Additive and Interaction Effects When Combined with Positive Controls. *Sci. Total Environ.* **2020**, *709*, 134490. https://doi.org/10.1016/j.scitotenv.2019.134490.

(7) Mostowy, S.; Boucontet, L.; Mazon Moya, M. J.; Sirianni, A.; Boudinot, P.; Hollinshead, M.; Cossart, P.; Herbomel, P.; Levraud, J.-P.; Colucci-Guyon, E. The Zebrafish as a New Model for the In Vivo Study of Shigella Flexneri Interaction with Phagocytes and Bacterial Autophagy. *PLoS Pathog.* **2013**, *9* (9), e1003588. https://doi.org/10.1371/journal.ppat.1003588.

(8) Maloney, E. M.; Taillebois, E.; Gilles, N.; Morrissey, C. A.; Liber, K.; Servent, D.; Thany, S. H. Binding Properties to Nicotinic Acetylcholine Receptors Can Explain Differential Toxicity of Neonicotinoid Insecticides in Chironomidae. *Aquat. Toxicol.* **2021**, *230*, 105701. https://doi.org/10.1016/j.aquatox.2020.105701.

(9) Janssen, E. M.-L.; Choi, Y.; Luthy, R. G. Assessment of Nontoxic, Secondary Effects of Sorbent Amendment to Sediments on the Deposit-Feeding Organism Neanthes Arenaceodentata. *Environ. Sci. Technol.* **2012**, *46* (7), 4134–4141. https://doi.org/10.1021/es204066g.

(10) Zhao, Y.-X.; Jiang, H.-Y.; Cheng, X.; Zhu, Y.-X.; Fan, Z.-X.; Dai, Z.-L.; Guo, L.; Liu, Z.-H.; Dai, Y.-J. Neonicotinoid Thiacloprid Transformation by the N2-Fixing Bacterium Microvirga Flocculans CGMCC 1.16731 and Toxicity of the Amide Metabolite. *Int. Biodeterior. Biodegrad.* **2019**, *145*, 104806. https://doi.org/10.1016/j.ibiod.2019.104806.

(11) Tennekes, H. A.; Sánchez-Bayo, F. The Molecular Basis of Simple Relationships between Exposure Concentration and Toxic Effects with Time. *Toxicology* **2013**, *309*, 39–51. https://doi.org/10.1016/j.tox.2013.04.007.

(12) Rösch, A.; Gottardi, M.; Vignet, C.; Cedergreen, N.; Hollender, J. Mechanistic Understanding of the Synergistic Potential of Azole Fungicides in the Aquatic Invertebrate Gammarus Pulex. *Environ. Sci. Technol.* **2017**, *51* (21), 12784–12795. https://doi.org/10.1021/acs.est.7b03088.

(13) Buchwalter, D. B.; Jenkins, J. J.; Curtis, L. R. Temperature Influences on Water Permeability and Chlorpyrifos Uptake in Aquatic Insects with Differing Respiratory Strategies. *Environ. Toxicol. Chem.* **2003**, *22* (11), 2806–2812. https://doi.org/10.1897/02-350.

(14) Saitoh, T.; Wennogle, L. P.; Changeux, J.-P. Factors Regulating the Susceptibility of the Acetylcholine Receptor Protein to Heat Inactivation. *FEBS Lett.* **1979**, *108* (2), 489–494.
